# Imbalance of Th17 cells, Treg cells and associated cytokines in patients with systemic lupus erythematosus: a meta-analysis

**DOI:** 10.3389/fimmu.2024.1425847

**Published:** 2024-07-17

**Authors:** Jinge Huang, Xiaolong Li, Qingmiao Zhu, Meijiao Wang, Zhijun Xie, Ting Zhao

**Affiliations:** ^1^ The First Affiliated Hospital of Zhejiang Chinese Medical University (Zhejiang Provincial Hospital of Chinese Medicine), Hangzhou, China; ^2^ College of Basic Medical Sciences, Zhejiang Chinese Medical University, Hangzhou, China; ^3^ Key Laboratory of Chinese Medicine Rheumatology of Zhejiang Province, Research Institute of Chinese Medical Clinical Foundation and Immunology, College of Basic Medical Science, Zhejiang Chinese Medical University, Hangzhou, China

**Keywords:** systemic lupus erythematosus, T helper 17 cell, regulatory T cell, cytokine, meta-analysis

## Abstract

**Objective:**

This article aims to investigate the changes of T helper 17 (Th17) cells, regulatory T (Treg) cells and their associated cytokines in patients with systemic lupus erythematosus (SLE).

**Methods:**

Multiple databases were investigated to identify articles that explored Th17 cells, Treg cells and relevant cytokines in SLE patients. A random effects model was used for calculating pooled standardized mean differences. Stata version 15.0 was utilized to conduct the meta-analysis.

**Results:**

The levels of Th17 cells, IL-17, IL-6, IL-21 and IL-10 were higher in SLE patients than in healthy controls (HCs), but the TGF-β levels were lower. The percentage of Treg cells was lower than HCs in SLE individuals older than 33. Among studies that had 93% or lower females, the percentage of Th17 cells was greater in patients than in HCs. However, the percentage of Treg cells was lower when the proportion of females was less than 90%. Patients with lupus nephritis or active SLE had an increased proportion of Th17 cells and a decreased proportion of Treg cells.

**Conclusions:**

The increased level of Th17 cells and related cytokines could be the main reason for the elevated Th17/Treg ratio in SLE. The percentages of Th17 and Treg cells were associated with gender, age, disease activity and kidney function. Furthermore, the reduced proportions of Treg cells may primarily result in a rise in the Th17/Treg ratio in older or active SLE patients.

**Systematic Review Registration:**

https://www.crd.york.ac.uk/prospero, identifier CRD42023454937.

## 1 Introduction

Systemic lupus erythematosus (SLE) is a chronic autoimmune disease characterized by immune cell activation and tissue inflammation, causing profound damage to multiple organs (1). The prevalence of SLE is about 9 times higher in females compared to males, with a mean age of 35 years (2). The etiology and pathogenesis of SLE are complex and accompanied by abnormal immune responses, such as dysfunction of T lymphocytes and disorder of CD4^+^ T lymphocytes (3). Moreover, accumulating evidence suggests that SLE is driven by the imbalances of lymphocyte subsets, especially the imbalance between T helper 17 (Th17) and regulatory T (Treg) cells, disrupting the equilibrium of the immune system (4–7).

Th17 cells are a subset of effector CD4^+^ T cells that have pro-inflammatory effects (8). In contrast, Treg cells with anti-inflammatory effects are essential to prevent the restoration of immunological homeostasis (9). Treg cells comprise a quite heterogeneous population and are either induced by conversion of conventional T cells existing in the periphery (iTregs) or are derived from developing T cells with intermediate self-reactivity in the thymus (nTregs) (10). The phenotype CD4^+^CD25^+^Foxp3^+^ is believed to better define nTregs, which make up approximately 80% of the Treg repertoire (11, 12). Although several researches have examined the levels of Th17 and CD4^+^CD25^+^Foxp3^+^Treg cells in SLE patients, many of these findings are contradictory. Most studies suggested that the percentages of Th17 cells were significantly increased in patients with SLE, yet a few studies found that Th17 cells might be decreased in the peripheral blood of SLE patients (13, 14). Similarly, some studies reported increased proportions of Treg cells, some studies reported no changes in Treg cells, and some studies reported reduced proportions of Treg cells (13, 15, 16). Furthermore, different studies have provided conflicting data regarding the imbalance of Th17 and Treg cells (17, 18). The reason could be connected to the proportion of women, age, disease activity, abnormal kidney function and medication use. Therefore, it is necessary to perform a meta-analysis to systematically evaluate changes in the proportions of Th17 and Treg cells in SLE patients.

Cytokines play important roles in regulating immune responses and different T lymphocytes synthesize different cytokines (19). Th17 cells secrete a profile of potent proinflammatory cytokines, including IL-17, IL-21 and IL-6, while Treg cells produce TGF-β and IL-10 (20). It has been reported that most of these cytokines have pro-inflammatory properties. For instance, IL-17-producing cells have been found in the kidneys of lupus-prone mice and patients with lupus nephritis (LN), suggesting that IL-17 contributes to organ damage (21). In addition, some cytokines have immunomodulatory or anti-inflammatory roles, such as IL-10 (22). Many cytokines are related to disease activity and represent promising detection indicators and therapeutic targets of SLE (23, 24). Nonetheless, contradicting results have been reported in studies investigating changes in cytokines in the serum of SLE patients. For example, Antiga et al. reported that TGF-β levels were lower in patients with SLE than in healthy people, while in the study of Xing Q et al., SLE patients had no changes in serum IL-6 compared with healthy people (25, 26).

The purpose of our study was to identify the changes in Th17 cells, Treg cells and their related cytokines in patients with SLE. Since there are many controversies in the research on the imbalance of Th17, Treg cells and relevant cytokines in SLE patients, our work would have a positive reference value to better understand the pathogenesis of this disease. This is a crucial step to improve clinical diagnosis and treatment strategies.

## 2 Methods

This study protocol was registered at PROSPERO (CRD42023454937). Preferred Reporting Items for Systematic Reviews and Meta-Analyses (PRISMA) guidelines were followed for all stages of design, implementation, analysis and reporting in this study (27).

### 2.1 Data sources and search strategy

A comprehensive literature search was conducted in PubMed, Embase, Web of Science, Cochrane Library, China National Knowledge Infrastructure (CNKI) and Wanfang Database without language limitation from their inception to August 15, 2023. The search strings consisted of 3 topics: SLE, Th17 cell and Treg cell. Both Medical Subject Headings (MeSH) terms and free text words were used to identify all potentially relevant articles. Additionally, a manual search of potentially relevant citations was conducted by using the references of the selected articles, relevant reviews and previous meta-analyses. The actual search strategy listing all search terms used and how they were combined is available in **Appendix A**.

### 2.2 Study selection criteria

Only studies fulfilling the following inclusion criteria were eligible to be included in this study: a) evaluated the proportion of Th17 cells and Treg cells or the levels of their associated cytokines in peripheral blood; b) included SLE patients diagnosed according to the American College of Rheumatology (ACR) criteria or the Systemic Lupus International Collaborating Clinics (SLICC) criteria; c) were designed as case-control studies; d) Treg cells were identified by the expression of CD4, CD25 and Foxp3; e) Th17 cells were identified by the expression of CD4 and IL-17; f) original data were expressed as or could be converted to mean and standard deviation (SD) (28, 29).

The exclusion criteria were the following: a) duplicate reports, insufficient data, or unpublished abstracts; b) studies recruited participants with other autoimmune conditions apart from SLE; c) cytokine levels were not measured in whole peripheral blood.

Two reviewers (Xiaolong Li and Qingmiao Zhu) independently screened relevant articles by titles or abstracts and, if necessary, full text. In case of discrepancies between the two reviewers, a third independent reviewer (Jinge Huang) arbitrated the decision.

### 2.3 Data extraction and methodological quality assessment

Data were independently extracted from eligible studies by two reviewers (Xiaolong Li and Qingmiao Zhu) using a predesigned extraction form. Any discrepancies were resolved through discussion or a re-checked by a third reviewer (Jinge Huang). The data extraction form comprised 4 parts covering the following variables: a) first author, year of publication, and country in which the study was performed; b) the characteristics of eligible studies, including patient population, percentage of women, average age and medication use; c) diagnosis criteria for SLE and experimental methods; d) mean percentage of Th17 and Treg cells, mean Th17/Treg ratio, and relevant cytokine levels secreted by SLE patients and healthy controls (HCs).

If the required information was missing, the original authors of the studies were contacted to gather complete and consistent study information. GetData Graph Digitizer version 2.25 (http://getdata-graph-digitizer.com/) was used to extract data from graphs (30). Given that each eligible article was an observational study, the Newcastle-Ottawa Scale (NOS) was adopted to assess the methodological quality of the included studies.

### 2.4 Statistical analysis

For continuous variables, we chose standardized mean differences (SMDs) as a measure of effect size. Pooled SMDs and 95% confidence intervals (CI) were calculated by applying a random effects model with restricted maximum likelihood (REML), as this model provided a more conservative estimate than the fixed-effect model by incorporating both within- and between-study variation (31). A difference with a p-value below 0.05 was considered statistically significant.

The degree of heterogeneity is among the major concerns in our meta-analysis, as non-homogeneous data may limit the reliability of conclusions. Firstly, heterogeneity was assessed by the Q-statistic test whereby an outcome with a *p*-value above 0.05 was considered statistically insignificant. Based on the Higgins classification, I^2^-values of 25%, 50% and 75% were defined as low, moderate and high heterogeneity, respectively (32). If the heterogeneity was high (I^2^>75%), further analysis encompassing subgroup analysis and sensitivity analysis was performed to examine the source of the heterogeneity. The Begg test and Egger test were applied to evaluate publication bias (33, 34). Statistical analyses were conducted using Stata version 15.0 with the package “meta”.

## 3 Results

### 3.1 Study selection and characteristics

**Figure 1:**
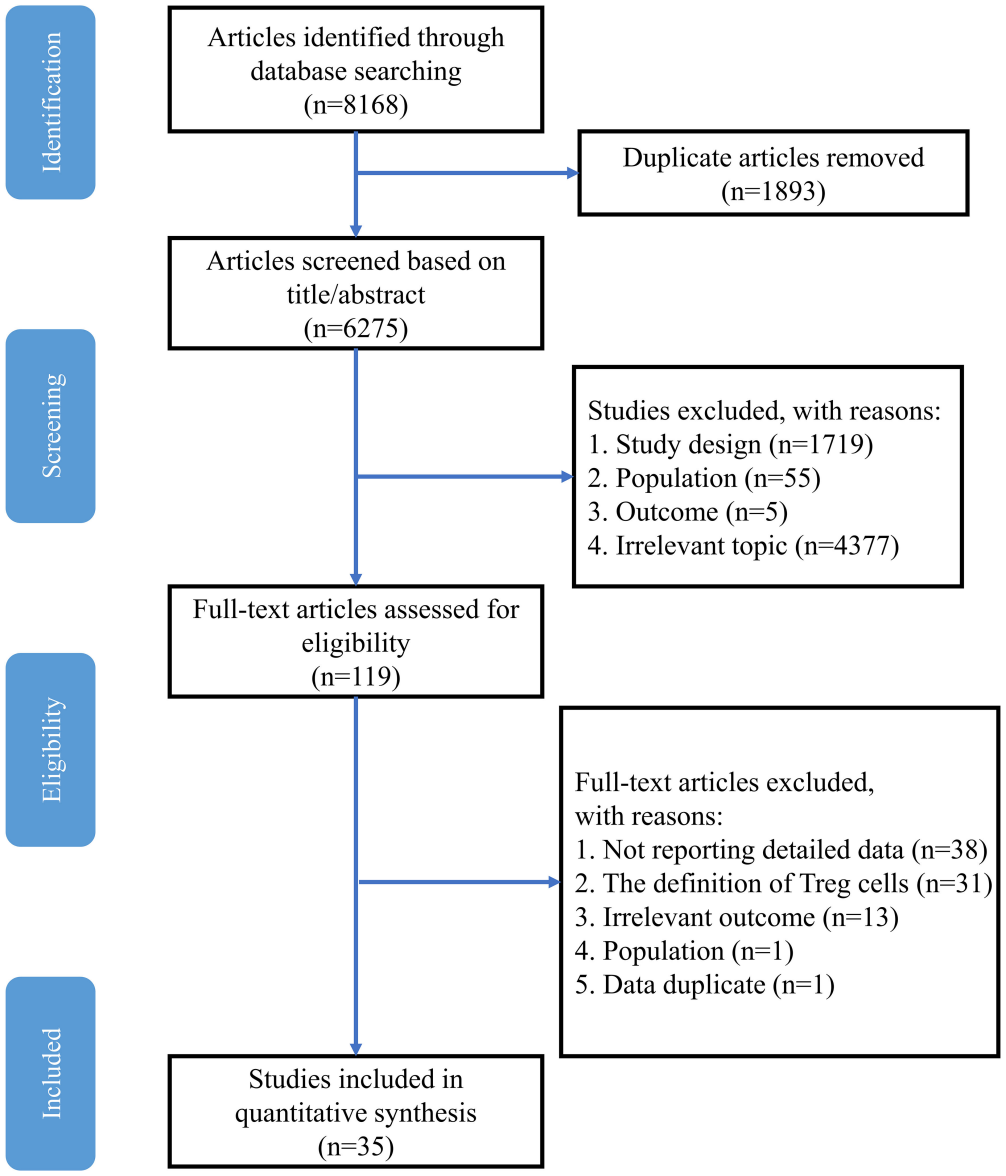
Study flow diagram.

A total of 8168 potentially eligible studies were retrieved from the electronic databases, and 6275 studies remained after removal of duplicates. In these records, 4377 were excluded due to irrelevant studies, and 1779 were excluded because of study design, population and outcome. Therefore, 119 records remained for full article screening, and 82 articles were removed due to insufficient data, Treg definition and irrelevant outcomes. Moreover, 2 studies were excluded because of data duplication and the wrong population. Finally, 35 studies were included in this meta-analysis. The specific process is shown in **Figure 1**. The detailed characteristics of the 35 studies included are presented in **Table 1**, and the NOS score varied between 6 and 9 in each included study (**Supplementary Table B.1**).

**Table 1:** Characteristics of included studies.

Study	Location	SLE case/HC case		SLE Case	Experimental methods	Outcomes
	Country	Number of Female Sex (%)	Age (Mean ± SD OR Mean)	Medication	Technique	
PENG Xuebiao (2012) (35)	China	42(95.2%)/20(95%)	27.48 ± 9.38/29.58 ± 13.95	No record of medication use	Flow cytometry; ELISA	Th17; Treg; Th17/Treg; IL-17
CAI Xiao-yan (2012) (36)	China	80(88.7%)/20(NA)	33.1052 ± 10.898/NA	No record of medication use	Flow cytometry	Th17; Treg; Th17/Treg
Che Guozhu (2015) (37)	China	103(86.4%)/30(NA)	33.04 ± 13.64/NA	Treatment with glucocorticoids	Flow cytometry	Th17; Treg; Th17/Treg
Cheng Chuanfang (2014) (38)	China	40(NA)/13(NA)	32.87 ± 12.88/31.6	No record of medication use	Flow cytometry	Th17; Treg; Th17/Treg
Hao Hui-qin (2018) (39)	China	100(89%)/30(73.3%)	35 ± 12/39 ± 10	No record of medication use	Flow cytometry; ELISA	Th17; Treg; Th17/Treg; IL-17; TGF-β
LI Zhi (2015) (40)	China	38(86.8%)/21(NA)	36.2 ± 12.51/40.3 ± 10.21	No record of medication use	Flow cytometry; ELISA	Th17; Treg; IL-17; TGF-β; IL-6
Luo Min (2011) (41)	China	50(90%)/20(NA)	34.3 ± 9.2/NA	No record of medication use	Flow cytometry	Th17; Treg; Th17/Treg
Zhao Lidan (2010) (42)	China	46(91.3%)/29(NA)	29.6 ± 10.7/NA	Treatment with glucocorticoids	Flow cytometry	Th17; Treg
Álvarez-Rodríguez L (2019) (5)	Spain	11(100%)/21(71.4%)	32.8 ± 13.1/40.3 ± 11.6	No use of glucocorticoids	Flow cytometry	Th17
Barath S (2007) (43)	Hungary	72(87.5%)/41(NA)	43.4 ± 13.9/NA	No use of glucocorticoids	Flow cytometry	Treg
Chen M (2018) (6)	China	39(100%)/33(NA)	28.82 ± 7.06/27.4 ± 7.78	No use of glucocorticoids	Flow cytometry; ELISA	Th17; Treg; Th17/Treg; IL-17
El-Maraghy N (2018) (15)	Egypt	56(100%)/30(100%)	27.8 ± 6.4/29.3 ± 5.8	No record of medication use	Flow cytometry	Treg
Fakhfakh R (2022) (44)	Tunisia	9(NA)/5(NA)	35 ± 10/30 ± 12	Treatment with glucocorticoids	Flow cytometry	Th17
Handono K (2016) (17)	Indonesia	62(100%)/62(100%)	31.2 ± 7.3/32.3 ± 6.3	No record of medication use	Flow cytometry	Th17; Treg; Th17/Treg
Henriques A (2010) (45)	Portugal	34(88.2%)/13(76.9%)	32 ± 11(active SLE); 34 ± 9(inactive SLE)/34 ± 10	Treatment with glucocorticoids	Flow cytometry	Th17
Kleczynska W (2011) (13)	Poland	15(93.3%)/11(73%)	41.5 ± 13.8/34.0 ± 10.2	Treatment with glucocorticoids	Flow cytometry	Th17; Treg
Li HT (2022) (4)	China	585(100%)/91(100%)	42.81 ± 12.19/42.82 ± 14.42	No record of medication use	Flow cytometry	Th17/Treg
Margiotta D (2016) (46)	Italy	13(100%)/11(100%)	45.3 ± 10.8/46.2 ± 8.3	Treatment with glucocorticoids	Flow cytometry	Th17
Robak E (2013) (47)	Poland	60(93.3%)/20(NA)	39.2/NA	Treatment with glucocorticoids	ELISA	IL-17
Shah K (2010) (48)	America	25(100%)/26(NA)	37.7 ± 11.4/NA	No use of glucocorticoids	Flow cytometry; ELISA	Treg; IL-6; IL-21; IL-10
Suen JL (2009) (49)	China	87(90.8%)/36(94.4%)	40 ± 11(active); 36 ± 11(inactive)/37 ± 12	Treatment with glucocorticoids	Flow cytometry	Treg
Talaat RM (2015) (20)	Egypt	60(93.3%)/24(91.7%)	46.3 ± 9.0/29.70 ± 6.96	Treatment with glucocorticoids	ELISA	IL-17; TGF-β; IL-6
Xing Q (2012) (26)	China	60(96.7%)/28(96.4%)	35.8 ± 7.2/36.2 ± 5.6	No use of glucocorticoids	Flow cytometry; ELISA	Th17; Treg; IL-17; TGF-β
Yin ZJ (2018) (16)	China	280(92.5%)/38(92.1%)	36.15 ± 11.86/42.89 ± 15.15	Treatment with glucocorticoids	Flow cytometry	Treg
Zhang B (2008) (50)	China	21(90.5%)/11(NA)	NA	Treatment with glucocorticoids	Flow cytometry	Treg
Zecevic L (2018) (51)	Bosnia; Herzegovina	55(92.7%)/25(92.0%)	32.8 ± 11.0/35.5 ± 14.9	No use of glucocorticoids	Flow cytometry; ELISA	Treg; IL-17
Chu Xiujie (2022) (52)	China	55(96%)/55(98%)	31.88 ± 12.49/28.50 ± 12.71	No use of glucocorticoids	Flow cytometry; ELISA	Th17; Treg; Th17/Treg; IL-17; IL-21; IL-10
Huang Hua (2014) (53)	China	70(88.6%)/30(90%)	39 ± 13/40 ± 9	No record of medication use	Flow cytometry; ELISA	Th17; Treg; IL-17; IL-6
Zheng Leting (2016) (54)	China	36(88.9%)/28(89.2%)	35 ± 9/32 ± 8	No use of glucocorticoids	Flow cytometry; ELISA	Th17; IL-17; IL-21
Chen Fengying (2011) (55)	China	56(75%)/28(89.2%)	33 ± 8/28 ± 11	No record of medication use	Flow cytometry; ELISA	Th17; IL-17; TGF-β; IL-21; IL-10
Zhang Shaoran (2011) (56)	China	103(89.3%)/28(71.4%)	35 ± 12/39 ± 10	No record of medication use	Flow cytometry; ELISA	Th17; Treg; Th17/Treg; IL-6
Ivanova (2015) (57)	Bulgaria	54(NA)/24(NA)	39.3 ± 13.1/NA	No record of medication use	Flow cytometry	TGF-β; IL-10
Antiga E (2011) (25)	Italy	15(86.7%)/20(55%)	37.4 ± 8.6/51.3 ± 11.7	No use of glucocorticoids	ELISA	TGF-β; IL-10
Chen XQ (2010) (18)	China	60(100%)/56(100%)	NA	No use of glucocorticoids	ELISA	IL-17
Yang XY (2013) (14)	China	65 (89.2%)/30 (83.3%)	34 ± 11/32 ± 10	Treatment with glucocorticoids	Flow cytometry; ELISA	Th17; IL-17

### 3.2 Changes in the proportions of Th17 and Treg cells in SLE patients

#### 3.2.1 Th17 cells

**Figure 2:**
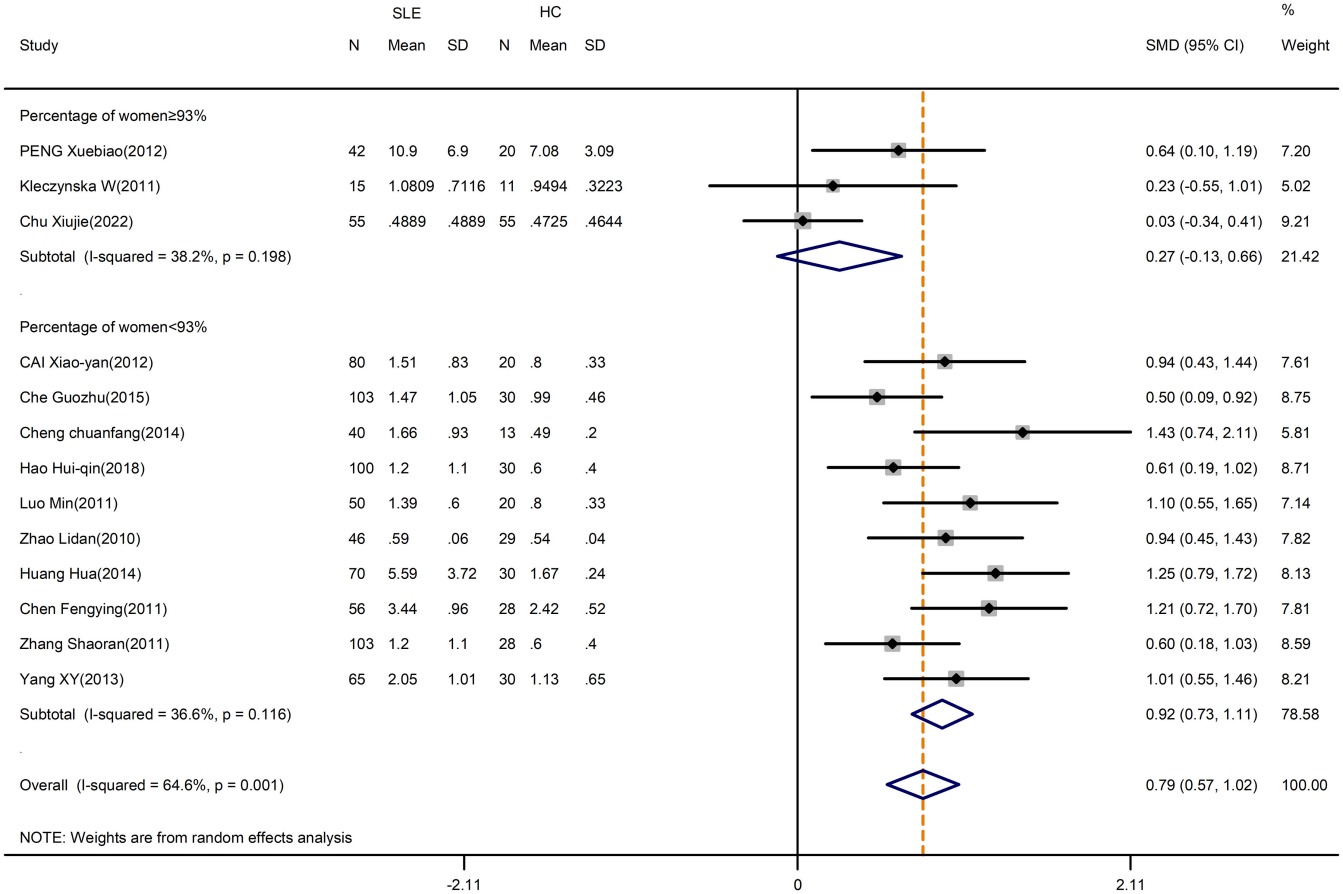
Subgroup analysis of Th17 cells according to the percentage of female SLE patients in the total patient population (Chen M (2018), Handono K (2016) and Álvarez-Rodríguez L (2019) were removed). SLE, systemic lupus erythematosus; HCs, healthy controls.

The meta-analysis showed that compared with HCs, the patients with SLE had significantly increased levels of percentage of Th17 cells (SMD=1.14; 95%CI=0.75,1.52; p<0.001; I^2^ = 89.0%; n=16; **Supplementary Figure D.1**). Sensitivity analysis excluding the Chen M study, the Handono K study and the Álvarez-Rodríguez L study reduced the heterogeneity of these results, and the proportion of Th17 cells remained higher in patients than HCs (SMD=0.79; 95%CI=0.57,1.02; p<0.001; I^2^ = 64.6%; n=13; **Figure 2**).

Since women and men had different SLE prevalence, a subgroup analysis was conducted to examine the effect of the proportion of women in the included study. The heterogeneity of the studies with more than 93% of women was reduced to 36.6% by subgroup analysis, but the overall results were unchanged (**Supplementary Figure D.1**). After excluding the Chen M study, the Handono K study and the Álvarez-Rodríguez L study, SLE patients had a greater change in the percentage of Th17 cells than HCs when the proportion of women was less than 93% (SMD=0.92; 95%CI=0.73,1.11; p<0.001; I^2^ = 36.6%; n=10; **Figure 2**; **Table 2**). However, the percentages of Th17 cells in patients were comparable to those in HCs when the proportion of women was greater than or equal to 93% (SMD=0.27; 95%CI=-0.13,0.66; p=0.182; I^2^ = 38.2%; n=3; **Figure 2**; **Table 2**). Thus, for studies in which the proportion of women was more than 93%, the proportions of women may introduce the high heterogeneity of the results.

**Table 2:** Subgroup analysis in Th17 cells according to the proportion of female patients in total patients and the use of GCs.

	Studies	SMD	*p-*value (%)	I^2^ (%)	p-Value for Heterogeneity	95% CI
Proportion of women						
aProportion≥ 93	3	0.27	0.182	38.2%	0.198	-0.13,0.66
Proportion<93	10	0.92	<0.001	36.6%	0.116	0.73,1.11
GCs use						
No record of medication use	9	1.08	<0.001	74.1%	<0.001	0.76,1.40
Treatment with GCs	4	0.72	<0.001	38.6%	0.181	0.40,1.05
No use of GCs	3	2.19	0.124	97.9%	<0.001	-0.60,4.99

GC, glucocorticoid.

In addition, a subgroup analysis by glucocorticoids (GCs) use indicated that medicated patients had a higher percentage of Th17 cells than HCs (SMD=0.72; 95%CI=0.40,1.05; p<0.001; n=4; **Supplementary Figure D.2**; **Table 2**).

#### 3.2.2 Treg cells

**Figure 3:**
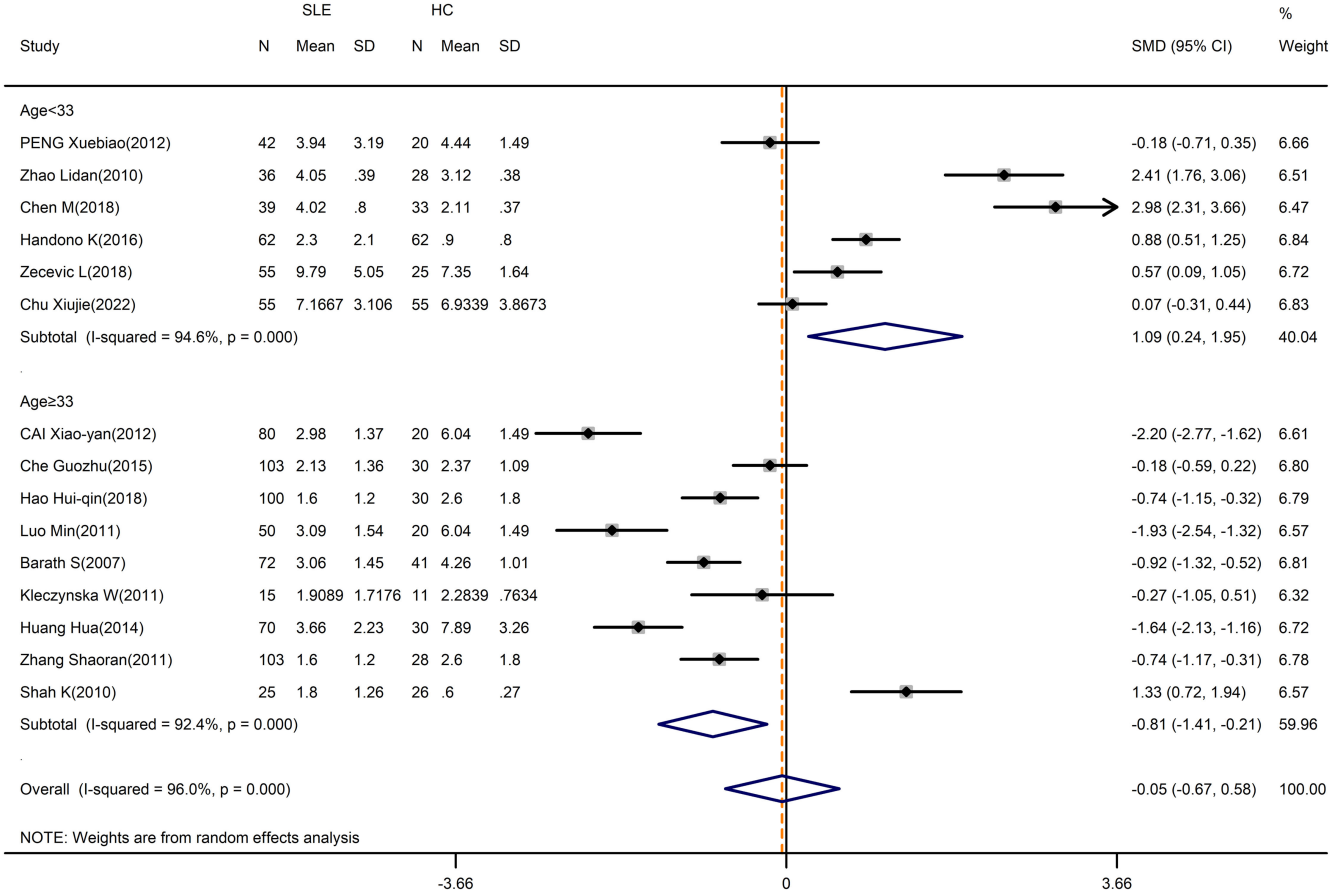
Subgroup analysis of Treg cells according to the patient age (Cheng chuanfang (2014), Yin ZJ (2018), and El-Maraghy N (2018) were removed). SLE, systemic lupus erythematosus; HCs, healthy controls.

Despite the high heterogeneity (I^2^ = 98.5%), no significant alteration was found when comparing the percentage of Treg cells in patients and HCs (SMD=-0.03; 95%CI=-1.09,1.02; p=0.951; n=18; **Supplementary Figure D.3**). Sensitivity analysis was conducted by excluding the Cheng chuanfang study, the Yin ZJ study and the El-Maraghy N study to reduce the heterogeneity of these results, but the overall results were not changed (SMD=-0.05; 95%CI=-0.67,0.58; p=0.886; n=15; **Figure 3**).

We performed a subgroup analysis by average age, with the exclusion of the Cheng chuanfang study, the Yin ZJ study and the El-Maraghy N study. Especially, the percentage of Treg cells in SLE patients who were less than 33 years old appeared to be higher than in HCs (SMD=1.09; 95%CI=0.24,1.95; p=0.012; n=6; **Figure 3**; **Table 3**), whereas the percentage of Treg cells in patients who were 33 years old or more was lower than HCs (SMD=-0.81; 95%CI=-1.41, -0.21; p=0.008; n=9; **Figure 3**; **Table 3**).

**Table 3:** Subgroup analysis in Treg cells according to the average age of patients, the proportion of female patients in total patients and the use of GCs.

	Studies	SMD	*p-*value (%)	I^2^ (%)	p-Value for Heterogeneity	95% CI
Age						
Age≥33	9	-0.81	0.008	92.4%	<0.001	-1.41, -0.21
Age<33	6	1.09	0.012	94.6%	<0.001	0.24,1.95
Proportion of women						
Proportion≥ 90	11	1.19	0.162	98.9%	<0.001	-0.48,2.85
Proportion<90	6	-1.05	<0.001	87.8%	<0.001	-1.57, -0.53
GCs use						
No record of medication use	9	-2.51	<0.001	97.6%	<0.001	-3.69, -1.33
Treatment with GCs	4	4.88	0.049	99.5%	<0.001	0.02,9.75
No use of GCs	5	0.78	0.174	96.3%	<0.001	-0.35,1.91

GC, glucocorticoid.

Moreover, a subgroup analysis of the proportion of women suggested that the percentage of Treg cells in SLE patients was strongly affected by the proportion of females. For studies where the proportion of females was less than 90%, the percentage of Treg cells was lower in patients than HCs (SMD=-1.05; 95%CI=-1.57, -0.53; p<0.001; n=6; **Supplementary Figure D.3**; **Table 3**). Nevertheless, the findings indicated no distinction between patients and HCs in studies with 90% or more females (SMD=1.19; 95%CI=-0.48,2.85; p=0.162; n=11; **Supplementary Figure D.3**; **Table 3**). Based on sensitive analysis, the results excluding the 3 studies were not changed (**Supplementary Figure D.4**).

while no obvious changes in the percentage of Treg cells between patients without the use of GCs and HCs (SMD=0.78; 95%CI=-0.35,1.91; p=0.174; n=5; **Supplementary Figure D.5**; **Table 3**).

#### 3.2.3 Disease activity and levels of Th17 and Treg cells

**Figure 4:**
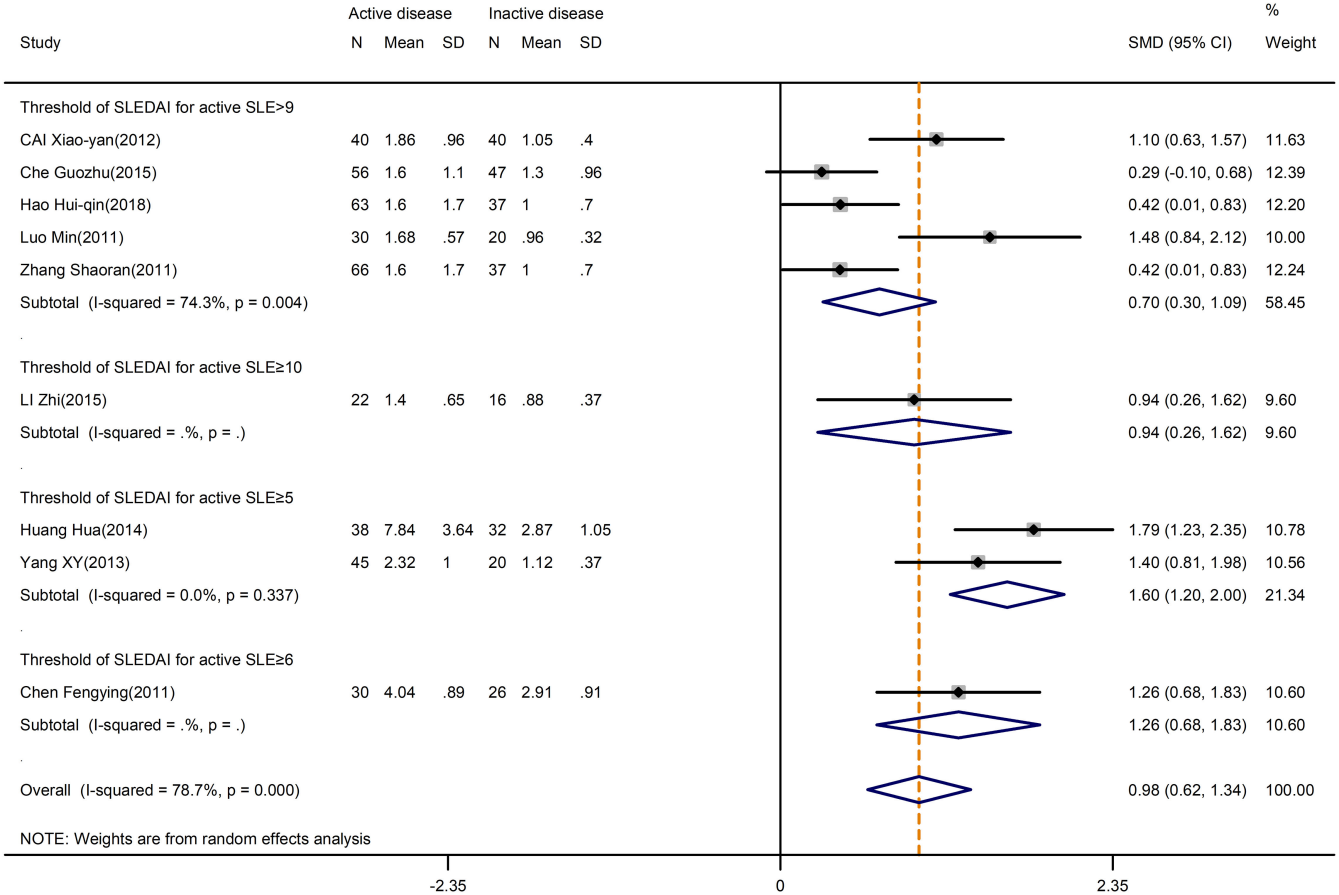
Subgroup analysis of Th17 cells according to the threshold of SLEDAI in active disease vs. inactive disease. SLEDAI, Systemic Lupus Erythematosus Disease Activity Index.

Firstly, our meta-analysis indicated disease activity had a significant relationship with the levels of Th17 cells. We observed a significant increase in the Th17 cells in active SLE compared to inactive SLE (SMD=0.98; 95%CI=0.62,1.34; p<0.001; n=9; **Figure 4**). Moreover, it seemed that there was no relationship between Th17 cells and the Systemic Lupus Erythematosus Disease Activity Index (SLEDAI) threshold, based on subgroup analysis by SLEDAI threshold chosen for the classification of active SLE (**Figure 4**).

**Figure 5:**
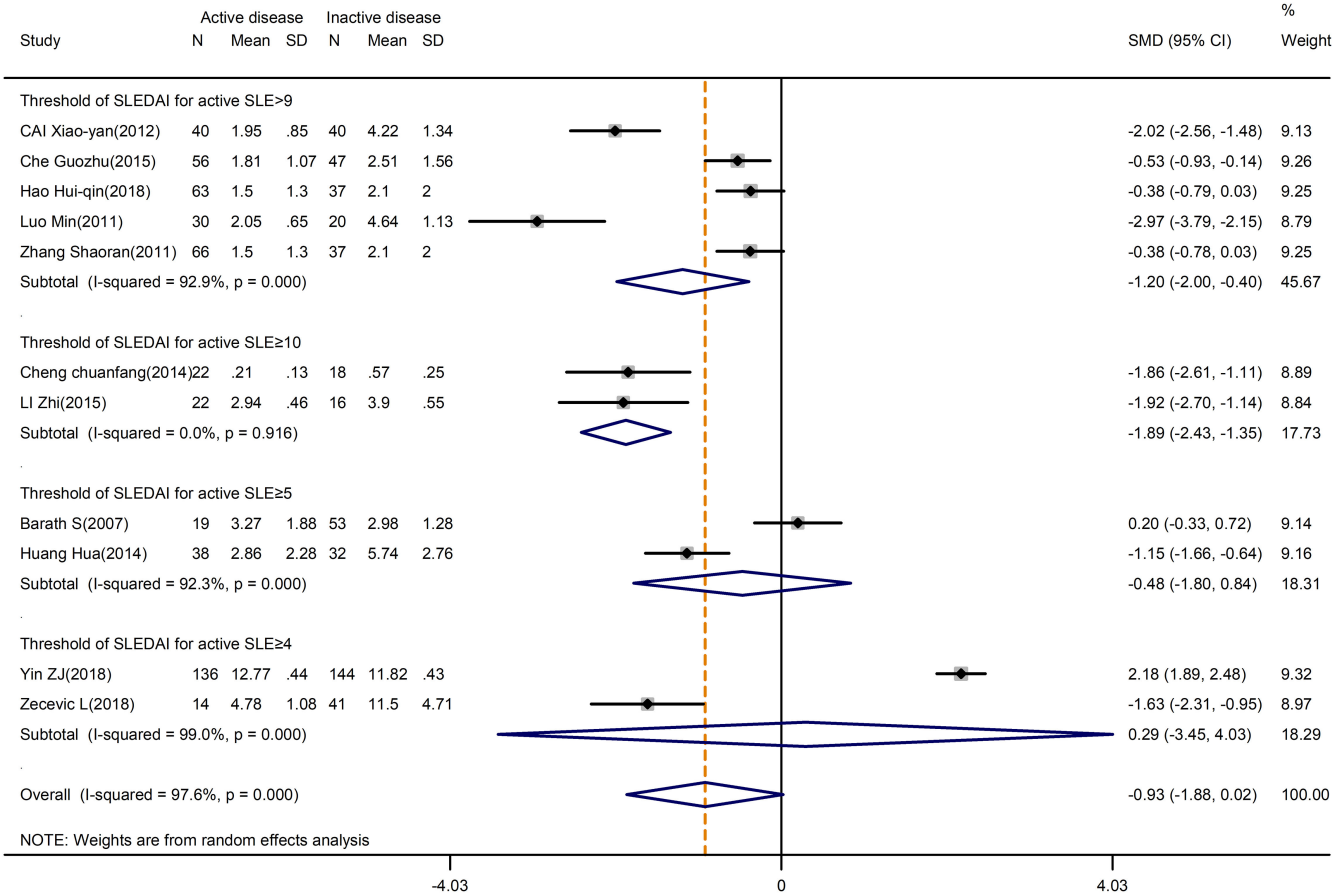
Subgroup analysis of Treg cells according to the threshold of SLEDAI in active disease vs. inactive disease. SLEDAI, Systemic Lupus Erythematosus Disease Activity Index.

On the other hand, the levels of Treg cells were not different in active and inactive SLE (SMD=-0.93; 95%CI=-1.88,0.02; p=0.879; n=11; **Figure 5**). We hypothesized that the threshold of SLEDAI chosen for active SLE definition might induce bias in the result. As expected, through subgroup analysis by the SLEDAI threshold, a significant difference in Treg levels was found in the studies that defined active SLE based on SLEDAI ≥9 (SMD=-1.20; 95%CI=-2.00, -0.40; p=0.003; n=5; **Figure 5**) and ≥10 (SMD=-1.89; 95%CI=-2.43, -1.35; p<0.001; n=2; **Figure 5**). Therefore, we speculate that the studies with a higher threshold of SLEDAI score tended to have a lower percentage of Tregs.

#### 3.2.4 Abnormal kidney function and levels of Th17 and Treg cells

One of the major organ manifestations of SLE is LN. The results showed that patients with abnormal renal function had a higher percentage of Th17 cells (SMD=1.38; 95%CI=0.14,2.62; p=0.029; n=5; **Supplementary Figure D.6**). Following sensitivity analysis, there was no apparent distinction between the two outcomes (**Supplementary Figure D.7**).

However, even though the overall analysis showed no difference in the proportion of Treg cells (SMD=0.43; 95%CI=-1,71,2.56; p=0.696; n=4; **Supplementary Figure D.8**), the analysis without the EI-Maraghy study indicated a lower percentage of Treg cells (SMD=-0.97; 95%CI=-1.77, -0.17; p=0.018; n=3; **Supplementary Figure D.9**) in patients with LN.

#### 3.2.5 Changes of the Th17/Treg ratio in SLE patients

**Figure 6:**
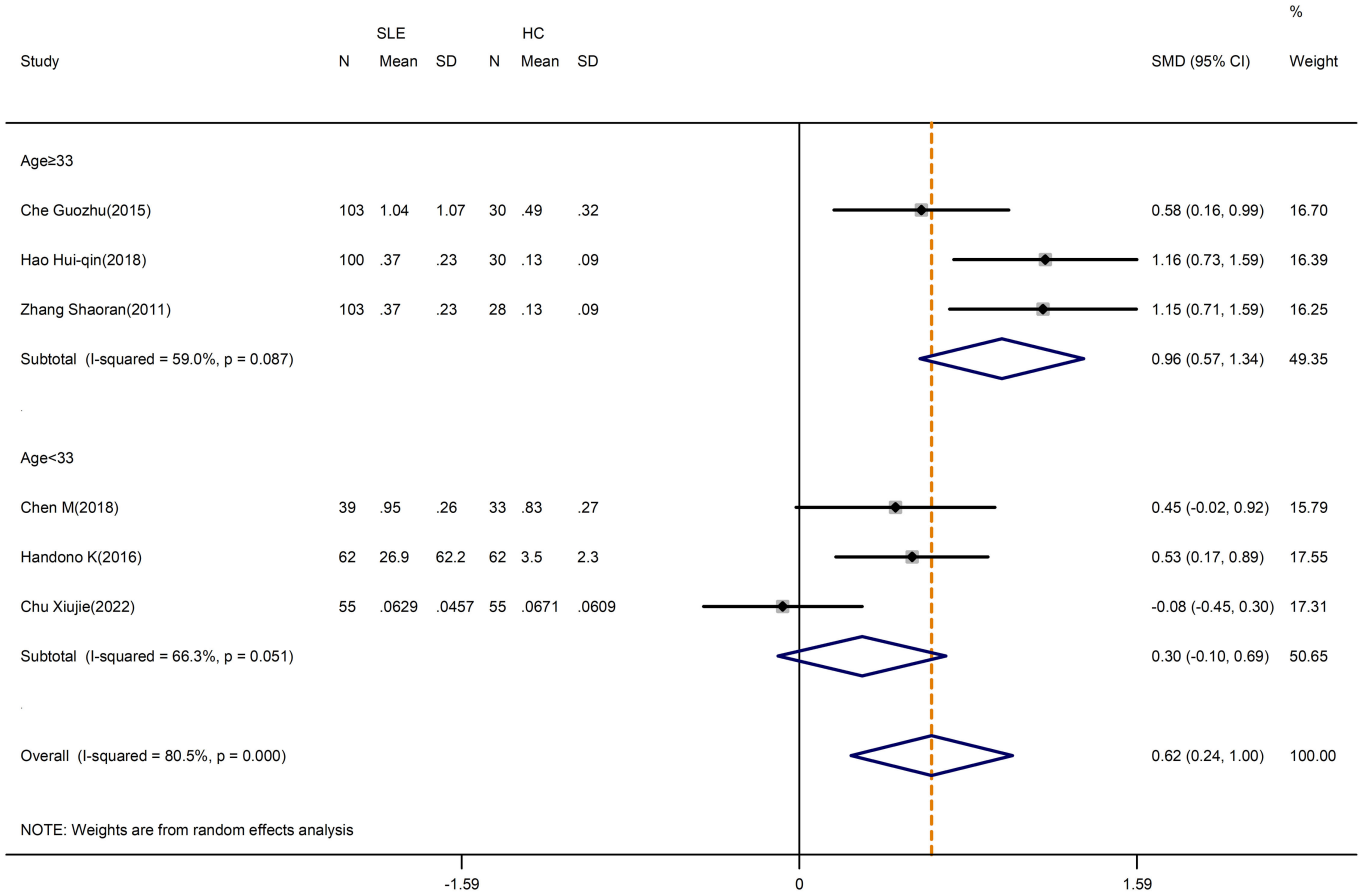
Subgroup analysis in the ratio change of Th17/Treg cells according to the patient age (Cheng chuanfang (2014) was removed). SLE, systemic lupus erythematosus; HCs, healthy controls.

The results of this analysis demonstrated that the Th17/Treg ratio was significantly higher in SLE patients than in HCs (SMD=0.80; 95%CI=0.36,1.24; p<0.001; n=7; **Supplementary Figure D.10**). Moreover, subgroup analysis based on average age reduced heterogeneity without altering the outcomes (**Supplementary Figure D.10**). Nevertheless, the Th17/Treg ratio in patients with an average age of less than 33 did not vary from HCs when the Cheng chuanfang research was eliminated using sensitivity analysis (SMD=0.30; 95%CI=-0.10,0.69; p=0.142; n=3; **Figure 6**).

**Figure 7:**
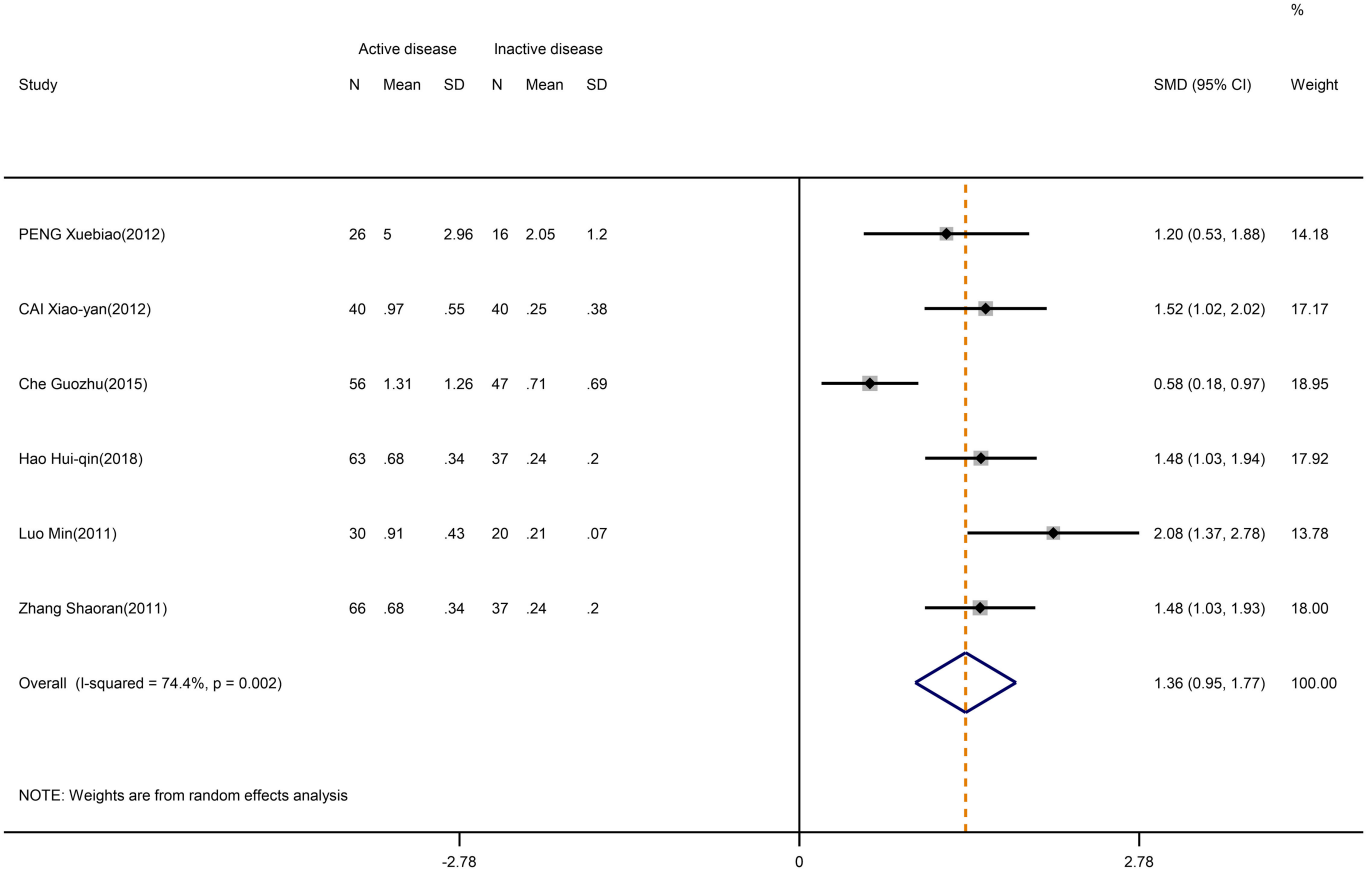
Forest plot of the ratio change of Th17/Treg cells in patients with SLE in active disease vs. inactive disease.

Furthermore, the Th17/Treg ratio was higher in active SLE than in inactive SLE (SMD=1.36; 95%CI=0.95,1.77; p<0.001; n=6; **Figure 7**). The results of the study showed no heterogeneity if the Che Guozhu study was removed by sensitivity analysis (**Supplementary Figure D.11**).

### 3.3 Variation of Th17 and Treg cytokines in SLE patients

The level of relevant cytokines in SLE patients compared with HCs was displayed in **Table 4**. A detailed meta-analysis of the different cytokines can be found in **Supplementary Figure D.12**-**21**.

**Table 4:** Meta-analysis of the level of relevant cytokines in SLE patients compared with HCs.

Cytokines	Studies	SMD	*p-*value (%)	I^2^ (%)	*p*-Value for Heterogeneity	95% CI
IL-17	11	1.17	<0.001	85.5	<0.001	0.66,1.34
IL-6	3	0.42	0.002	0	0.589	0.15,0.68
IL-21	4	2.42	0.005	97.0	<0.001	0.73,4.11
TGF-β	5	-0.92	0.001	83.5	<0.001	-1.48, -0.36
IL-10	5	0.49	0.002	43.1	0.134	0.19,0.79

#### 3.3.1 Th17 cytokines

**Figure 8:**
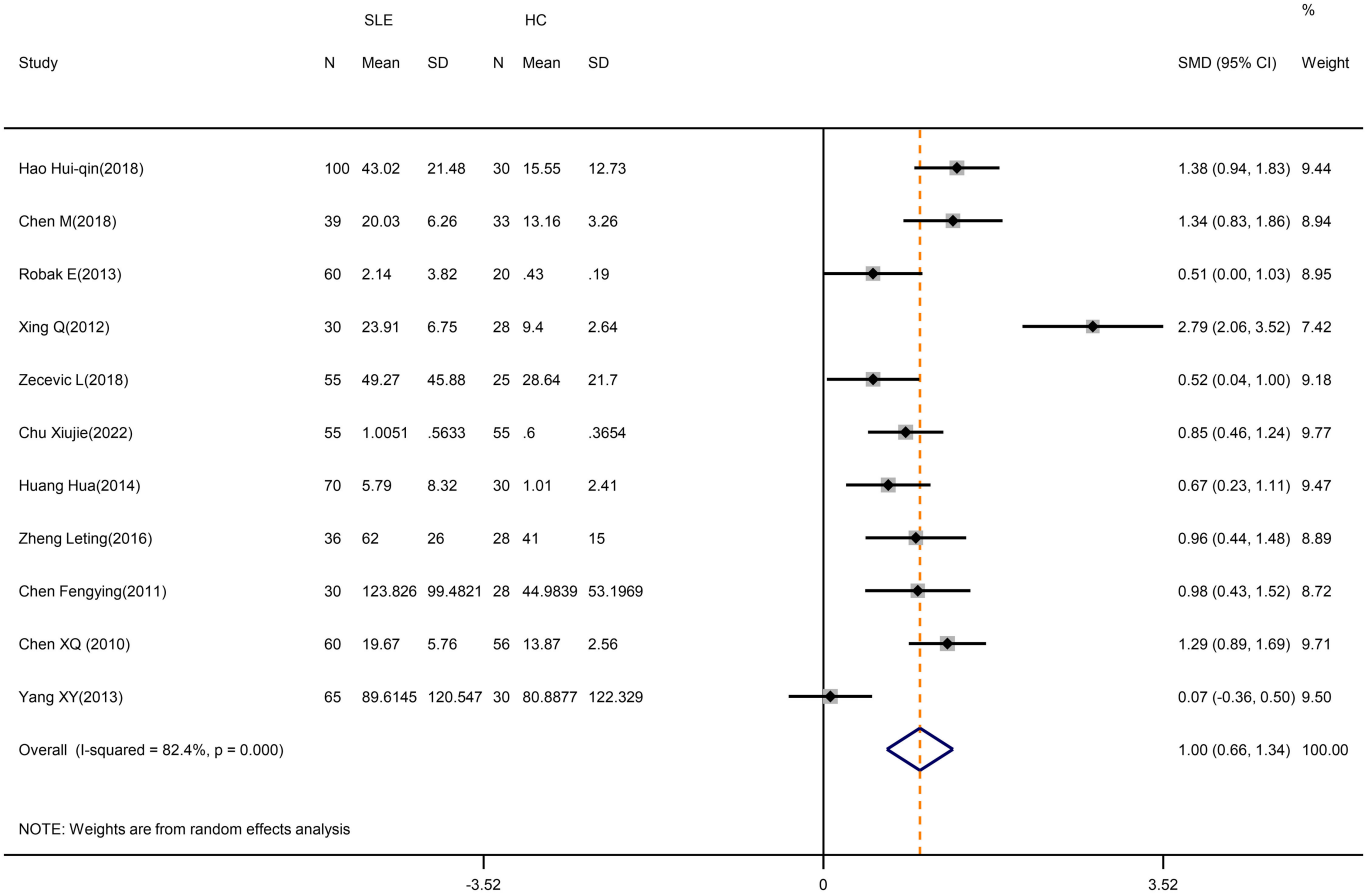
Forest plot of the level of IL-17 in SLE patients compared with HCs. SLE, systemic lupus erythematosus; HCs, healthy controls.

**Figure 9:**
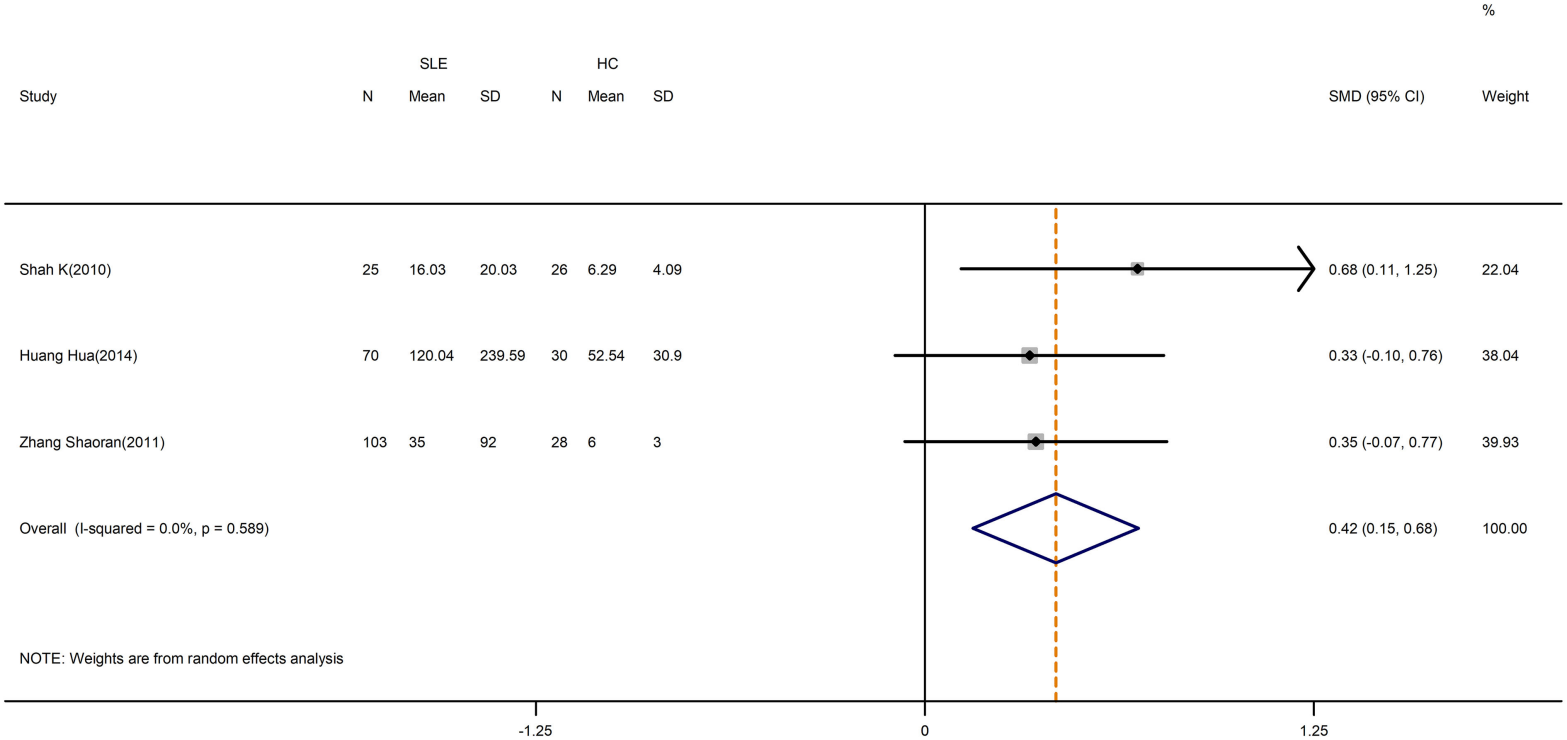
Forest plot of the level of IL-6 in SLE patients compared with HCs. SLE, systemic lupus erythematosus; HCs, healthy controls.

According to our meta-analysis, SLE patients exhibited greater levels of IL-17 (SMD=1.17; 95%CI=0.66,1.34; p<0.001; n=11; **Figure 8**), IL-21(SMD=2.42; 95%CI=0.73,4.11; p=0.005; n=4; **Supplementary Figure D.12**), and IL-6 (SMD=0.42; 95%CI=0.15,0.68; p=0.002; n=3; **Figure 9**) than HCs. Sensitivity analyses did not substantially alter these results (**Supplementary Figure D.13**,**14**). Meanwhile, the levels of IL-17 (SMD=0.84; 95%CI=0.43,1.25; p=0.003; n=7; **Table 5**; **Supplementary Figure D.15**, **16**) and IL-6 (SMD=2.12; 95%CI=0.21,4.02; p<0.001; n=3; **Table 5**; **Supplementary Figure D.17**) were greater in active SLE as compared to inactive SLE.

**Table 5:** Meta-analysis of the level change of relevant cytokines in active SLE compared with inactive SLE.

Cytokines	Studies	SMD	*p-*value (%)	I^2^ (%)	*p*-Value for Heterogeneity	95% CI
IL-17	7	0.84	<0.001	69.3	0.003	0.43,1.25
IL-6	3	2.12	0.030	95.9	<0.001	0.21,4.02
TGF-β	4	-0.17	0.760	92.7	<0.001	-1.26,0.92

#### 3.3.2 Treg cytokines

**Figure 10:**
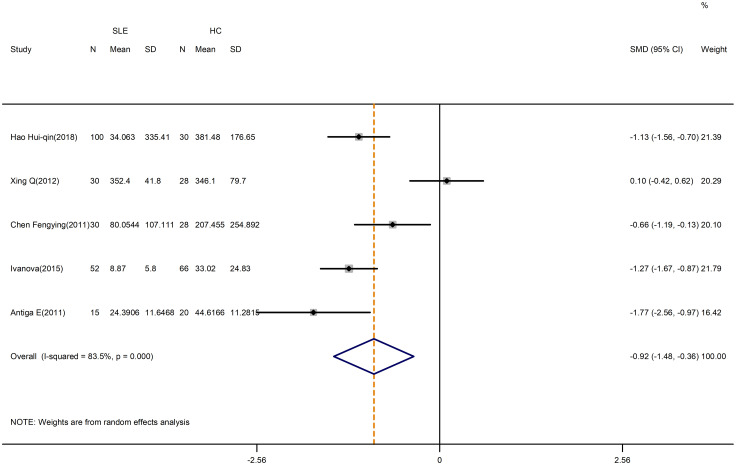
Forest plot of the level of TGF-β in SLE patients compared with HCs. SLE, systemic lupus erythematosus; HCs, healthy controls.

A pooled analysis of 5 studies revealed a significant decrease in the level of TGF-β in SLE patients compared to controls (SMD=-0.92; 95%CI=-1.48, -0.36; p=0.001; n=5; **Figure 10**). Despite that, the level of TGF-β in active SLE was comparable to those in the inactive SLE (SMD=-0.17; 95%CI=-1.26, 0.92; p=0.760; n=4; **Table 5**; **Supplementary Figure D.18**). Besides, IL-10 level was higher in patients with SLE versus HCs (SMD=0.49; 95%CI=0.19, 0.79; p=0.002; n=5; **Table 4**; **Supplementary Figure D.19**). The above results were not meaningfully affected when relevant studies were excluded from the sensitivity analysis (**Supplementary Figure D.20**, **21**).

### 3.4 Analysis of publication bias

The risk of publication bias was evaluated by using the Egger test and Begg test (**Supplementary Table C.1**). In addition to some evidence of biased publication regarding the percentage of Treg cells between active and inactive SLE, the results show a lack of evidence of publication bias in most studies, supporting robust stability.

## 4 Discussion

Although the imbalance of Th17 and Treg cells is currently considered to play a key role in the pathogenesis of SLE, studies to date have reported conflicting results (58). By summarizing 35 studies and 2617 SLE patients, our study has confirmed that the patients with SLE had increased levels of Th17 cells, Th17/Treg ratio, IL-17, IL-21, IL-6 and IL-10, but decreased levels of TGF-β. Furthermore, despite that there was no difference in the percentage of Treg cells between patients and HCs, our findings indicated that it was greatly influenced by age, sex ratio and medications.

Th17 cells that produce the major cytokine IL-17 have a dominant effector and pro-inflammatory functional profile. Previous studies indicated the involvement of the Th17/IL-17 axis in the pathogenesis of chronic autoimmune diseases (59). There is important evidence showing that IL-17 has a pleiotropic role in SLE pathology, including defending against pathogen infections, promoting the recruitment of neutrophils and other immune cells, and inducing the production of pro-inflammatory cytokines (60). In two distinct lupus animal models, there was an increase in the levels of IL-17 and Th17 cells (61). Moreover, Lee SY et al. illustrated that IL-17 genetic deletion could improve the pathology of SLE (62). Our meta-analysis showed that the percentage of Th17 cells and IL-17 levels were higher in SLE patients than in HCs. This implies that Th17 cells may promote the chronic inflammation of SLE by increasing the production of IL-17.

Apart from IL-17, Th17 cells also secrete other important cytokines and require certain cytokines for differentiation, such as IL-21 and IL-6. Regarding IL-21, it is not only expressed by Th17 cells but also involved in the initiation phase of Th17 cell differentiation (63). Notably, IL-21 cooperates with IL-17 to stimulate an inflammatory response and generates tissue damage in SLE (64). In the meanwhile, IL-6 is known to drive the expansion of Th17 cells, suggesting that the IL-17/IL-6 axis induces a positive feedback loop in SLE (65). Our meta-analysis substantiated that the levels of IL-21 and IL-6 were significantly elevated, which is also supported by the study of Vincent FB et al. (66). Hence, we supposed that these results may add more concrete evidence to the critical role Th17 cells play in the pathogenesis of SLE.

Treg cells, which are characterized by the expression of the transcription factor Foxp3, are a group of unique T lymphocyte subsets with a suppressive function that inhibits the excessive activation of self-antigens, monitors the expansion of lymphocytes, and effectively suppresses excessive immune responses (12, 67). A previous meta-analysis, which was published in 2018, demonstrated that there was a lower proportion of CD4^+^CD25^+^Foxp3^+^Treg cells in SLE patients than controls (68). Nevertheless, our results showed that there was no significant alteration in the proportions of Treg cells overall. Interestingly, we discovered the effect of age on Treg cells. Previous studies suggested that since the expression of the pro-apoptotic protein Bim in Treg cells was selectively lost with age, Treg levels were higher in the elderly than in the young (69). However, our subgroup analysis by average age revealed that Treg cells were more common in SLE patients with a mean age of less than 33, while they were less prevalent in those with a mean age of above 33. This may be attributed to thymic involution during aging, resulting in a decreased generation of Treg cells (70).

Furthermore, Treg cells can release the inhibitory cytokines TGF-β and IL-10 to exert immune-controlling and anti-inflammatory effects (71). TGF-β promotes the generation of Treg cells and plays both direct and indirect roles in Foxp3 expression (72). In our meta-analysis, SLE patients produce lower levels of TGF-β when compared with healthy individuals, which is also supported by the study of Becker-Merok et al. (73). Previous research found that high concentrations of TGF-β alone trigger the generation of Foxp3+ Treg cells, whereas low concentrations of TGF-β synergize with IL-6 to promote Th17-cell differentiation (72). Therefore, we hypothesized that this might be one of the reasons for the imbalance of Treg and Th17 cells.

In relation to IL-10, although it was generally considered an anti-inflammatory factor, our findings showed that patients had significantly higher levels of IL-10 than HCs, which is the opposite of the percentage of Treg cells. Recent reviews reported that IFN-α imparted the proinflammatory function of IL-10 to enhance inflammation in the development of SLE (74). Moreover, Saraiva et al. offered further proof that IL-10 had conflicting context-specific stimulatory and anti-inflammatory effects (75). These findings demonstrated that the dual function of IL-10 may be the cause of increased IL-10 levels in SLE patients. Additionally, the role of IL-10 in SLE calls for further research as it originates from multiple sources (76).

Besides, metabolic disorders of Th17 and Treg cells are crucial for the etiology and management of SLE. Energy metabolism is regulated by several signaling pathways, including mammalian target of rapamycin (mTOR)/adenosine 5’-monophosphate (AMP)-activated protein kinase (AMPK) signaling (77). Kato H et al. discovered that IL-21 activated mTOR complexes 1 and 2, and this in turn disrupted Treg cell autophagy, differentiation, and function (78). It suggests that Treg dysfunction in SLE is caused by IL-21-driven mTOR activation, which is a pharmacologically targetable checkpoint of deficient autophagy (78). Furthermore, drugs with clear clinical effects on SLE have been reported to alleviate the disordered energy metabolism of immune cells, indicating that energy metabolism may be targeted for SLE treatment. For instance, Lai ZW et al. demonstrated that sirolimus, a mTOR inhibitor, could enhance disease activity and control the rectification of pro-inflammatory T-cell lineage (79). Sun F et al. have shown that Metformin reduced disease flares by regulating the Th17/Treg balance through the AMPK/mTOR pathway (80).

In our study, disease activity had a significant association with the levels of Th17 cells, IL-17 and IL-6, but had no evident relationship with the levels of Treg cells and TGF-β. This result indicated that increased levels of IL-17 and IL-6 lead to a higher proportion of Th17 in patients with active SLE, which confirmed the reliability of earlier findings (81). For Treg cells, the proportion of Treg cells seemed to be influenced by the SLEDAI threshold used to differentiate between active and inactive SLE, regardless of the overall analysis showing no significant connection between Treg and activity disease. Notably, the higher threshold of the SLEDAI score tended to obtain a lower proportion of Treg cells. Given that the SLEDAI score may be positively correlated with the severity of SLE, Treg appears to be inversely connected with disease activity in SLE.

LN is one of the strongest predictors of a poor outcome in SLE, which is observed in approximately 50% of SLE patients. Our study illustrated that SLE patients with abnormal kidney function had a higher proportion of Th17 cells than patients with normal kidney function. Th17 cells have IL-17 as the main cytokines with receptors expressed in most intrinsic kidney cells. For instance, the Th17/IL-17 axis promotes the activation of profibrotic pathways with a consequent increase of extracellular matrix proteins in renal tubular epithelial cells (82). Besides, in the podocytes, the Th17/IL-17 axis leads to changes in the cytoskeleton with increased motility, reduced expression of healthy proteins, and increased oxidative stress (82). Thereby, the Th17/IL-17 axis can contribute to immune imbalance and LN. In contrast, the percentage of Treg cells was lower in patients with abnormal kidney function, implying an imbalanced Th17/Treg ratio in LN. IL-2 was found to be a master regulator of Treg cells in LN by Venkatadri R et al. (83). Likewise, recent research has demonstrated that the levels of IL-2R signaling in Tregs were reduced in inflammation, which further diminishes their suppressive function (84).

There is an intriguing finding from our study that using GCs can increase the proportion of Treg cells. Prior studies proved that GC signaling enhances the differentiation and function of Treg cells by up-regulating TGF-β receptors, Foxp3 and IL-10. On the other hand, our research revealed a higher level of Th17 cells in SLE patients receiving GCs, which was supported by prior research (85, 86). Banuelos et al. and de Castro Kroner et al. found that GC increased IL-17 production and RORγt expression, suggesting that GC allowed and even facilitated the Th17 reaction (85, 86).

It should be mentioned that imbalances in the proportion of Th17 and Treg cells may be related to disease characteristics in male SLE patients. In SLE populations with a larger percentage of males, the proportion of Th17 cells was higher and the proportion of Treg cells was lower. Recent research reported that males with SLE tend to have more severe disease with higher rates of renal and cardiovascular involvement (87). Our research corroborated that Th17 cells were more prevalent and Treg cells were less common in SLE patients with LN. Furthermore, Zhu et al. claimed that Th17/Treg imbalance may play a role in the formation and development of atherosclerosis (88). Thus, these discoveries might assist to explain why the Th17/Treg imbalance is more acute in males.

Alterations in the Th17/Treg ratio reinforced the above conclusions regarding the imbalance between Th17 and Treg cells in SLE and the potential underlying reasons. As to the overall analysis, the increased Th17/Treg ratio could primarily be caused by the increased Th17 cell levels. However, subgroup studies of age in SLE patients with an average age of less than 33 years revealed no significant changes in Th17/Treg, which is possibly because Treg cell levels are inversely correlated with age. In addition, we discovered that the Th17/Treg ratio was influenced by disease activity. Based on our results, we surmised that higher Th17 levels were the main source of this higher ratio. With the increase of the SLEDAI score, the decrease in Treg cell level also resulted in the increase of the Th17/Treg ratio.

However, there are additional important limitations in the evidence of the imbalance between Th17 and Treg cells in SLE, which need to be addressed. First, given the retrospective nature of studies mainly included, the statistical combination of data is subjected to a certain degree of selection and reporting biases. Specifically, according to the Egger test and Begg test, we identified a statistically significant publication bias in studies that evaluate the proportion of Treg cells between active and inactive SLE patients. Second, we were unable to perform subgroup analyses of relevant cytokines to explore plausible causes of between-study heterogeneity because of the insufficient number of studies. Moreover, even though we performed subgroup analyses of some results, it was not feasible to completely eliminate the heterogeneity across the studies due to the numerous additional variables that might have an impact on the results. Furthermore, analysis of SLE activity using different assessment tool, such as the British Isles Lupus Assessment Group (BILAG) index was not carried out due to the dearth of pertinent data. Finally, as the emphasis of our study was on the imbalance between Th17 and CD4^+^CD25^+^Foxp3^+^Treg cells, analysis of the balance connection between Th17 and Treg cells with other different phenotypes was not carried out. Because of the limitations of the current study, future well-designed prospective studies with large patient cohorts should be needed with the aim of clarifying the mentioned issues.

## 5 Conclusion

This meta-analysis found that the increased levels of Th17 cells and associated cytokines might be the primary cause for the elevated Th17/Treg ratio in SLE. There is substantial evidence that gender, age, treatments, disease activity and LN are strongly associated with the percentages of Th17 as well as Treg cells. Furthermore, the decreased proportions of Treg cells may primarily result in an increase in the Th17/Treg ratio in older or active SLE patients.

## Data availability statement

The original contributions presented in the study are included in the article/**Supplementary Material**. Further inquiries can be directed to the corresponding authors.

## Author contributions

JH: Writing – original draft. XL: Writing – original draft. QZ: Writing – original draft. MW: Methodology, Writing – review & editing. ZX: Methodology, Writing – review & editing. TZ: Methodology, Writing – review & editing.
