# Supplementary material for: Imbalance of Th17 cells, Treg cells and associated cytokines in patients with systemic lupus erythematosus: a meta-analysis
Source: Front Immunol. 2024 Jul 17;15:1425847. doi: 10.3389/fimmu.2024.1425847 (PMC11288813; doi:10.3389/fimmu.2024.1425847)
Supplement: Supplementary file 1 [file DataSheet_1.docx]

**Appendix A. Search strategy and Study flow diagram**

The search strategies of Pubmed and Web of Science were as follows. According to the characteristics of each database, the search strategy was adjusted to individual database constraints.

**Pubmed**

(("Lupus Erythematosus, Systemic"[Mesh]) OR (((((Systemic Lupus Erythematosus) OR (Lupus Erythematosus Disseminatus)) OR (Libman-Sacks Disease)) OR (Disease, Libman-Sacks)) OR (Libman Sacks Disease))) AND (((((((((((((((((((((((((((((((((((((("T-Lymphocytes, Regulatory"[Mesh]) OR (T Lymphocytes, Regulatory)) OR (Regulatory T-Lymphocyte)) OR (Regulatory T Lymphocyte)) OR (T-Lymphocyte, Regulatory)) OR (Treg Cell)) OR (Cell, Treg)) OR (Cells, Treg)) OR (Regulatory T-Lymphocytes)) OR (Regulatory T Lymphocytes)) OR (T-Cells, Regulatory)) OR (Treg Cells)) OR (Regulatory T-Cells)) OR (Regulatory T Cell)) OR (Cell, Regulatory T)) OR (Cells, Regulatory T)) OR (Regulatory T Cells)) OR (Th3 Cells)) OR (Cell, Th3)) OR (Cells, Th3)) OR (Th3 Cell)) OR (Suppressor T-Lymphocytes, Naturally-Occurring)) OR (Naturally-Occurring Suppressor T-Lymphocyte)) OR (Naturally-Occurring Suppressor T-Lymphocytes)) OR (Suppressor T Lymphocytes, Naturally Occurring)) OR (Suppressor T-Lymphocyte, Naturally-Occurring)) OR (Suppressor T-Cells, Naturally-Occurring)) OR (Naturally-Occurring Suppressor T-Cell)) OR (Naturally-Occurring Suppressor T-Cells)) OR (Suppressor T Cells, Naturally Occurring)) OR (Suppressor T-Cell, Naturally-Occurring)) OR (T-Cell, Naturally-Occurring Suppressor)) OR (T-Cells, Naturally-Occurring Suppressor)) OR (Tr1 Cells)) OR (Tr1 Cell)) OR (Cell, Tr1)) OR (Cells, Tr1)) OR ((((((((((((("Th17 Cells"[Mesh]) OR (Th17 Cell)) OR (Cell, Th17)) OR (Cells, Th17)) OR (T Helper 17 Cell)) OR (TH-17 Cell)) OR (T Helper 17 Cells)) OR (Type 17 Helper T Cells)) OR (TH-17 Cells)) OR (Cell, TH-17)) OR (Cells, TH-17)) OR (TH 17 Cells)) OR (Type 17 Helper T Cell)))

**Web of Science**

#1. Th17 Cells (Topic) or Th17 Cell (Topic) or Cell, Th17 (Topic) or Cells, Th17 (Topic) or T Helper 17 Cell (Topic) or TH-17 Cell (Topic) or TH 17 Cell (Topic) or T Helper 17 Cells (Topic) or Type 17 Helper T Cells (Topic) or TH-17 Cells (Topic) or Cell, TH-17 (Topic) or Cells, TH-17 (Topic) or TH 17 Cells (Topic) or Type 17 Helper T Cell (Topic)

#2. T-Lymphocytes, Regulatory (Topic) or T Lymphocytes, Regulatory (Topic) or Regulatory T-Lymphocyte (Topic) or Regulatory T Lymphocyte (Topic) or T-Lymphocyte, Regulatory (Topic) or Treg Cell (Topic) or Cell, Treg (Topic) or Cells, Treg (Topic) or Regulatory T-Lymphocytes (Topic) or Regulatory T Lymphocytes (Topic) or Treg Cells (Topic) or Regulatory T-Cells (Topic) or Regulatory T Cell (Topic) or Cell, Regulatory T (Topic) or Cells, Regulatory T (Topic) or Regulatory T Cells (Topic) or T Cell, Regulatory (Topic) or T Cells, Regulatory (Topic) or Regulatory T-Cell (Topic) or Th3 Cells (Topic) or Cell, Th3 (Topic) or Cells, Th3 (Topic) or Th3 Cell (Topic) or Suppressor T-Lymphocytes, Naturally-Occurring (Topic) or Naturally-Occurring Suppressor T-Lymphocyte (Topic) or Naturally-Occurring Suppressor T-Lymphocytes (Topic) or Suppressor T Lymphocytes, Naturally Occurring (Topic) or Suppressor T-Lymphocyte, Naturally-Occurring (Topic) or Suppressor T-Cells, Naturally-Occurring (Topic) or Naturally-Occurring Suppressor T-Cell (Topic) or Naturally-Occurring Suppressor T-Cells (Topic) or Suppressor T Cells, Naturally Occurring (Topic) or Suppressor T-Cell, Naturally-Occurring (Topic) or T-Cell, Naturally-Occurring Suppressor (Topic) or T-Cells, Naturally-Occurring Suppressor (Topic) or Tr1 Cells (Topic) or Tr1 Cell (Topic) or Cell, Tr1 (Topic) or Cells, Tr1 (Topic)

#3. Lupus Erythematosus, Systemic (Topic) or Systemic Lupus Erythematosus (Topic) or Lupus Erythematosus Disseminatus (Topic) or Libman-Sacks Disease (Topic) or Disease, Libman-Sacks (Topic) or Libman Sacks Disease (Topic)

#4. #1 or #2

#5. #3 and #4
