# Supplementary material for: Imbalance of Th17 cells, Treg cells and associated cytokines in patients with systemic lupus erythematosus: a meta-analysis
Source: Front Immunol. 2024 Jul 17;15:1425847. doi: 10.3389/fimmu.2024.1425847 (PMC11288813; doi:10.3389/fimmu.2024.1425847)
Supplement: Supplementary file 2 [file DataSheet_2.docx]

Appendix B. Quality assessment of each included study

Table B.1. Bias risk assessment with reference to the Newcastle Ottawa Scale (NOS)

| Study | **Selection** | | | | **Comparability** | **Exposure** | | | **Total score** |
| --- | --- | --- | --- | --- | --- | --- | --- | --- | --- |
|  | Adequate Definition of Cases | Representativeness of the Cases | Selection of Controls | Definition of Controls | Comparability of Cases and Controls on the Basis of the Design or Analysis | Ascertainment of Exposure | Same method of ascertainment for cases and controls | Non-Response Rate |  |
| PENG Xuebiao (2012) | 1 | 1 | 1 | 1 | 2 | 1 | 1 | 0 | 8 |
| CAI Xiao-yan (2012) | 1 | 1 | 1 | 1 | 2 | 1 | 1 | 0 | 8 |
| Che Guozhu (2015) | 1 | 1 | 1 | 0 | 2 | 1 | 1 | 0 | 7 |
| Cheng chuanfang (2014) | 1 | 1 | 1 | 1 | 2 | 1 | 1 | 0 | 8 |
| Hao Hui-qin (2018) | 1 | 1 | 1 | 1 | 2 | 1 | 1 | 0 | 8 |
| LI Zhi (2015) | 1 | 1 | 1 | 1 | 1 | 1 | 1 | 0 | 7 |
| Luo Min (2011) | 1 | 1 | 0 | 1 | 2 | 1 | 1 | 0 | 8 |
| Zhao Lidan (2010) | 1 | 1 | 1 | 1 | 2 | 1 | 1 | 0 | 8 |
| Álvarez-Rodríguez L (2019) | 1 | 1 | 1 | 1 | 2 | 1 | 1 | 0 | 8 |
| Barath S (2007) | 1 | 1 | 0 | 1 | 2 | 1 | 1 | 0 | 7 |
| Chen M (2018) | 1 | 1 | 0 | 1 | 2 | 1 | 1 | 0 | 7 |
| El-Maraghy N (2018) | 1 | 1 | 0 | 1 | 1 | 1 | 1 | 0 | 6 |
| Fakhfakh R (2022) | 1 | 1 | 0 | 1 | 1 | 1 | 1 | 0 | 6 |
| Handono K (2016) | 1 | 1 | 0 | 1 | 2 | 1 | 1 | 0 | 7 |
| Henriques A (2010) | 1 | 1 | 1 | 1 | 2 | 1 | 1 | 0 | 8 |
| Kleczynska W (2011) | 1 | 1 | 1 | 1 | 1 | 1 | 1 | 0 | 7 |
| Li HT (2022) | 1 | 1 | 1 | 1 | 1 | 1 | 1 | 0 | 7 |
| Margiotta D (2016) | 1 | 1 | 1 | 1 | 1 | 1 | 1 | 0 | 7 |
| Robak E (2013) | 1 | 1 | 1 | 1 | 2 | 1 | 1 | 0 | 8 |
| Shah K (2010) | 1 | 1 | 1 | 1 | 2 | 1 | 1 | 0 | 8 |
| Suen JL (2009) | 1 | 1 | 1 | 1 | 2 | 1 | 1 | 0 | 8 |
| Talaat RM (2015) | 1 | 1 | 1 | 1 | 2 | 1 | 1 | 0 | 8 |
| Xing Q (2012) | 1 | 1 | 1 | 1 | 1 | 1 | 1 | 0 | 7 |
| Yin ZJ (2018) | 1 | 1 | 1 | 1 | 2 | 1 | 1 | 0 | 8 |
| Zhang B (2008) | 1 | 1 | 1 | 1 | 2 | 1 | 1 | 0 | 8 |
| Zecevic L (2018) | 1 | 1 | 1 | 1 | 2 | 1 | 1 | 0 | 8 |
| Chu Xiujie (2022) | 1 | 1 | 1 | 1 | 2 | 1 | 1 | 0 | 8 |
| Huang Hua (2014) | 1 | 1 | 1 | 1 | 1 | 1 | 1 | 0 | 7 |
| Zheng Leting (2016) | 1 | 1 | 1 | 1 | 2 | 1 | 1 | 0 | 8 |
| Chen Fengying (2011) | 1 | 1 | 1 | 1 | 2 | 1 | 1 | 0 | 8 |
| Zhang Shaoran (2011) | 1 | 1 | 1 | 1 | 2 | 1 | 1 | 0 | 8 |
| Ivanova (2015) | 1 | 1 | 1 | 1 | 1 | 1 | 1 | 0 | 7 |
| Antiga E (2011) | 1 | 1 | 1 | 1 | 2 | 1 | 1 | 0 | 8 |
| Chen XQ (2010) | 1 | 1 | 1 | 1 | 2 | 1 | 1 | 0 | 8 |
| Yang XY (2013) | 1 | 1 | 1 | 1 | 2 | 1 | 1 | 0 | 8 |
