# Supplementary material for: Imbalance of Th17 cells, Treg cells and associated cytokines in patients with systemic lupus erythematosus: a meta-analysis
Source: Front Immunol. 2024 Jul 17;15:1425847. doi: 10.3389/fimmu.2024.1425847 (PMC11288813; doi:10.3389/fimmu.2024.1425847)
Supplement: Supplementary file 3 [file DataSheet_3.docx]

Appendix C. Analysis of Publication Bias

Table C.1 Egger test and Begg test identifying the publication bias

|  | P>\|t\|(bias) | |
| --- | --- | --- |
|  | **Egger test** | **Begg test** |
| **T cells** |  |  |
| Th17 cell | 0.151 | 0.161 |
| Treg cell | 0.351 | 0.685 |
| **Disease activity** |  |  |
| Th17 cell | 0.007 | 0.048 |
| Treg cell | 0.002 | 0.02 |
| IL-17 | 0.193 | 0.266 |
| IL-6 | 0.148 | 0.296 |
| TGF-β | 0.664 | 0.734 |
| **kidney function** |  |  |
| Th17 cell | 0.731 | 0.462 |
| Treg cell | 0.071 | 0.308 |
| **Cytokines** |  |  |
| IL-17 | 0.13 | 0.276 |
| IL-6 | 0.09 | 1 |
| IL-21 | 0.088 | 0.308 |
| TGF-β | 0.995 | 1 |
| IL-10 | 0.206 | 0.462 |
