# Supplementary material for: Imbalance of Th17 cells, Treg cells and associated cytokines in patients with systemic lupus erythematosus: a meta-analysis
Source: Front Immunol. 2024 Jul 17;15:1425847. doi: 10.3389/fimmu.2024.1425847 (PMC11288813; doi:10.3389/fimmu.2024.1425847)
Supplement: Supplementary file 4 [file DataSheet_4.docx]

Appendix D. Forest plots of each meta-analysis

**
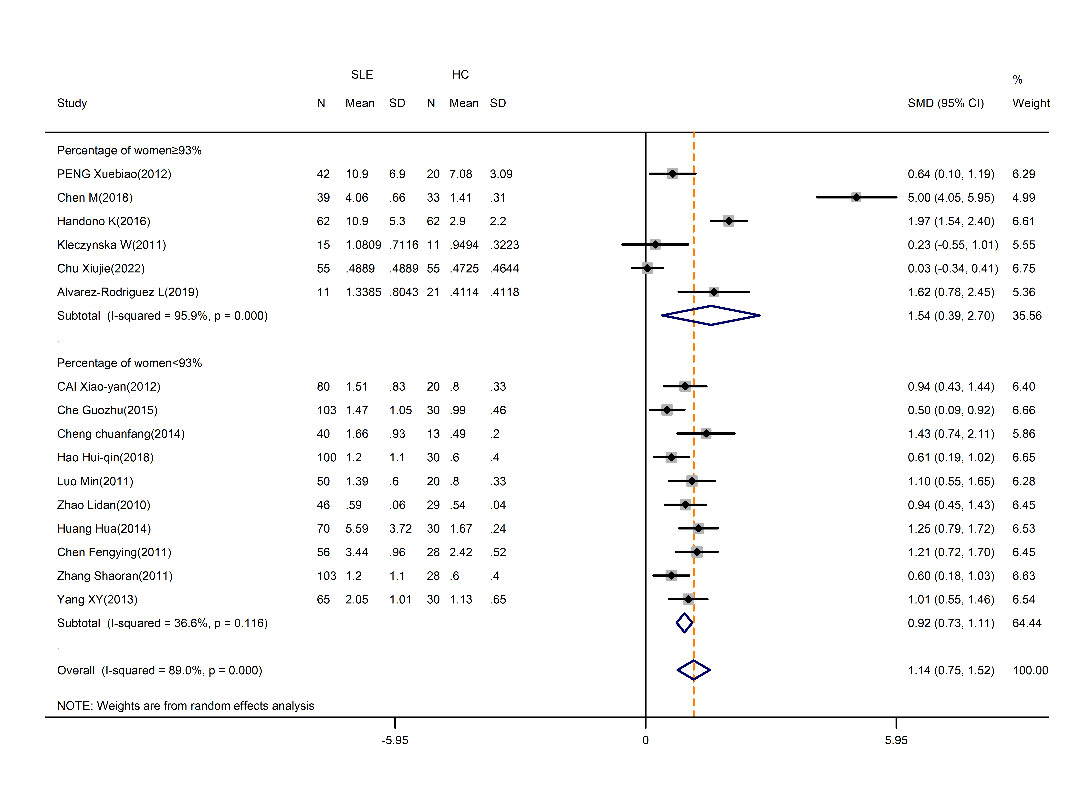
**

Supplementary Fig.D. 1 Subgroup analysis of Th17 cells according to the percentage of female SLE patients in the total patient population.


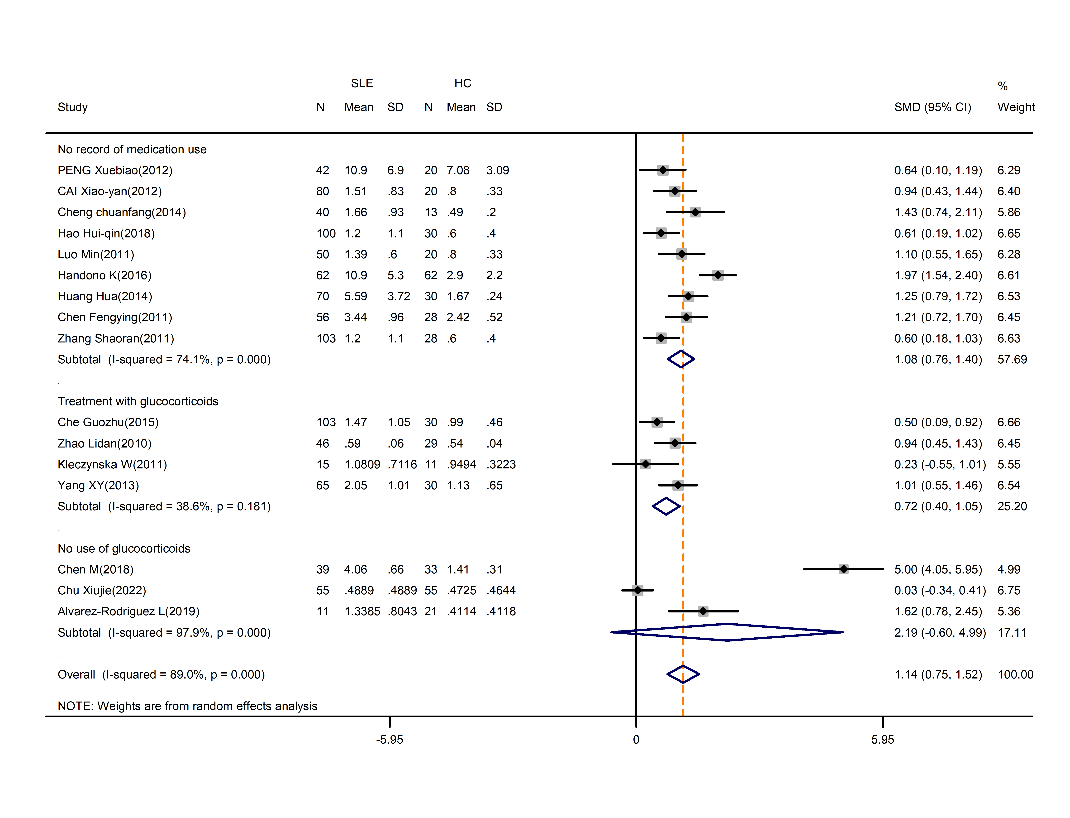


Supplementary Fig.D. 2 Subgroup analysis of Th17 cells according to glucocorticoid use.


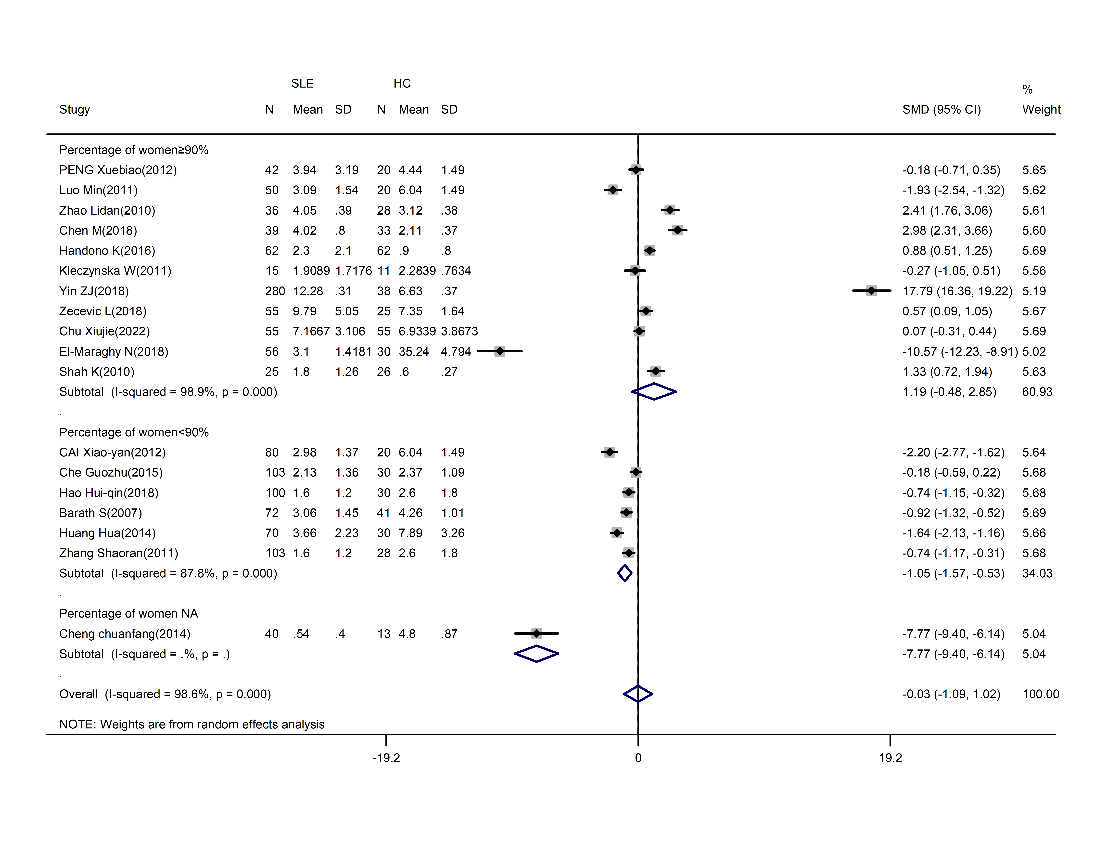


Supplementary Fig.D. 3 Subgroup analysis of Treg cells according to the percentage of female SLE patients in the total patient population.

Supplementary Fig.D. 4 Subgroup analysis of Treg cells according to the percentage of female SLE patients in the total patient population (Cheng chuanfang (2014), Yin ZJ (2018), and El-Maraghy N (2018) were removed).


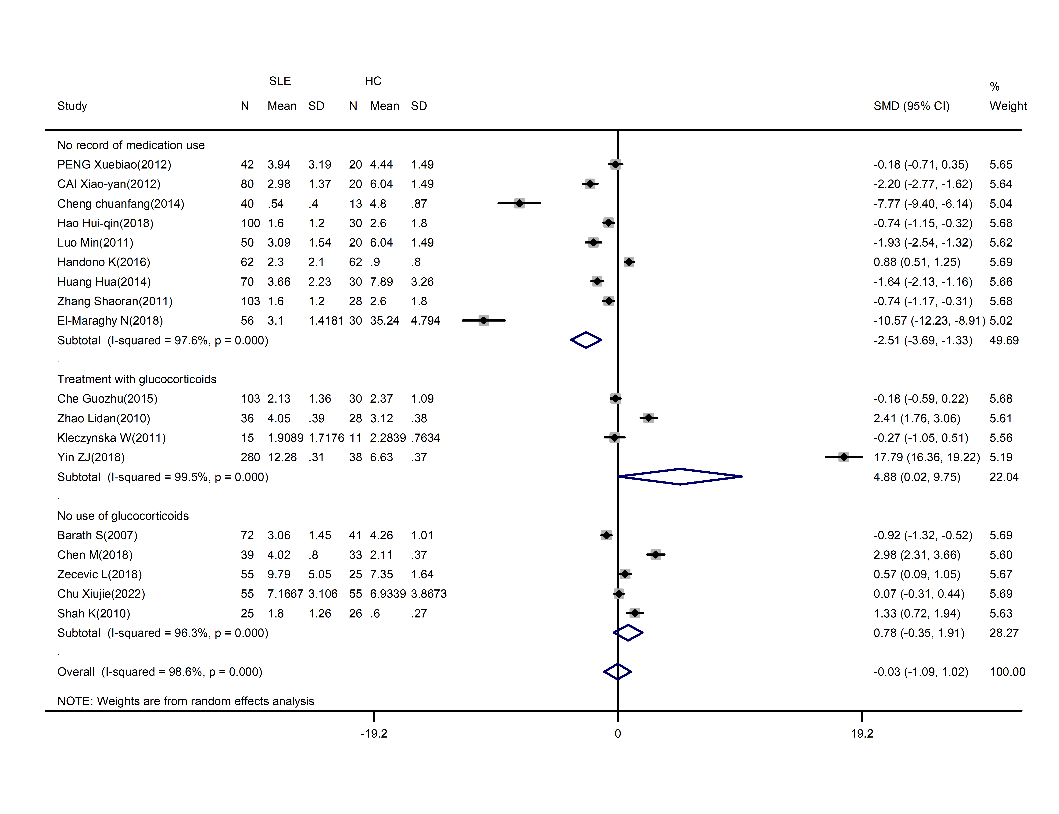


Supplementary Fig.D. 5 Subgroup analysis of Treg cells according to glucocorticoid use.


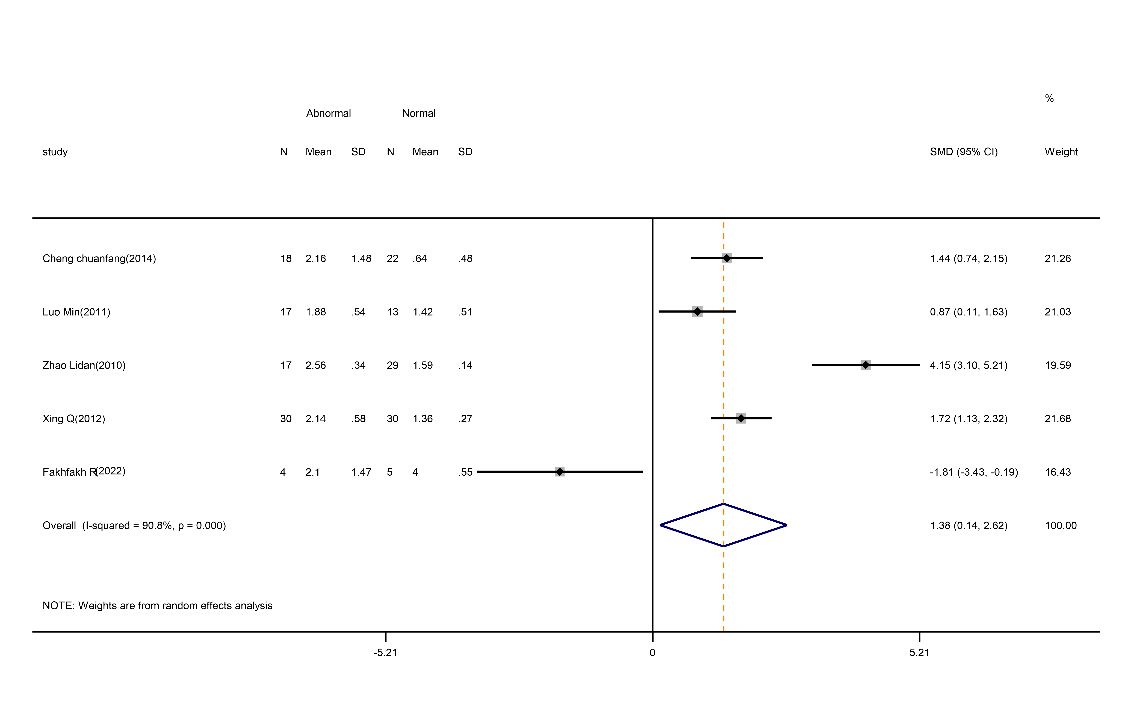


Supplementary Fig.D. 6 Subgroup analysis in Th17 cells according to kidney function of SLE patients. Abnormal: SLE patients with abnormal kidney function. Normal: SLE patients with normal kidney function.


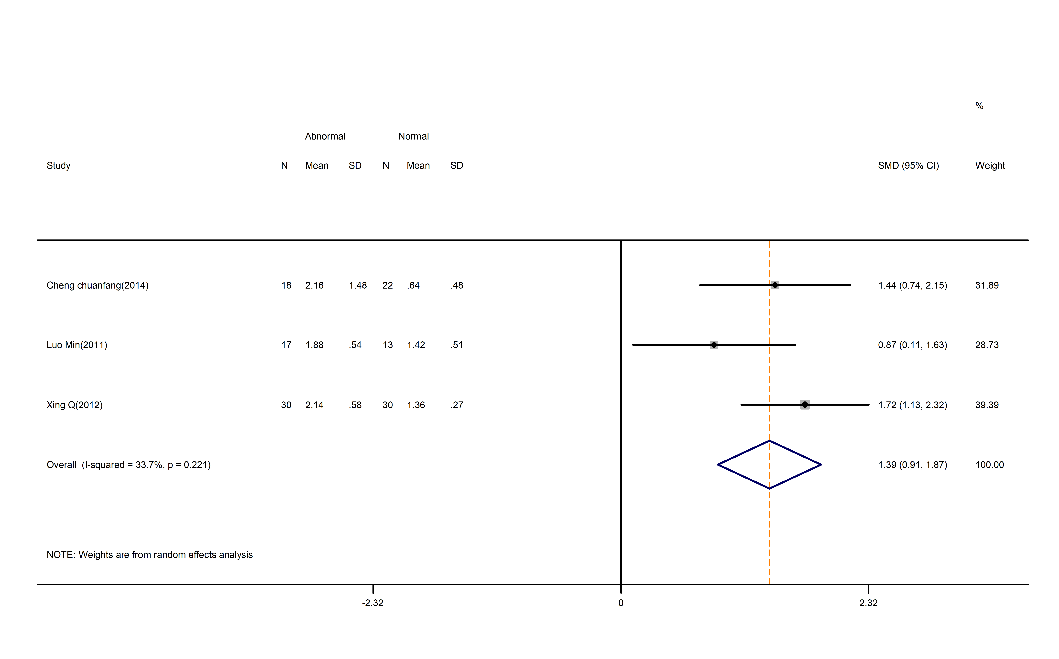


Supplementary Fig.D. 7 Subgroup analysis in Th17 cells according to kidney function of SLE patients. Abnormal: SLE patients with abnormal kidney function (Zhao Lidan (2010) and Fakhfakh R (2022) were removed). Normal: SLE patients with normal kidney function.


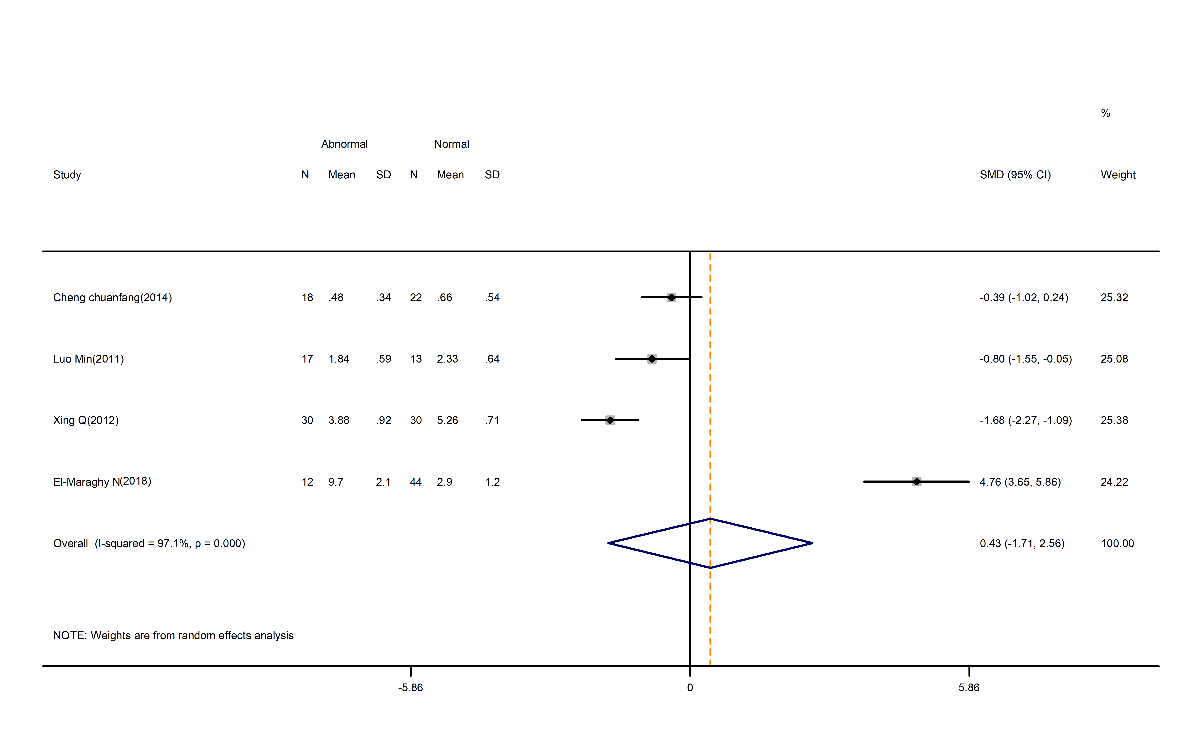


Supplementary Fig.D. 8 Subgroup analysis in Treg cells according to kidney function of SLE patients. Abnormal: SLE patients with abnormal kidney function. Normal: SLE patients with normal kidney function.


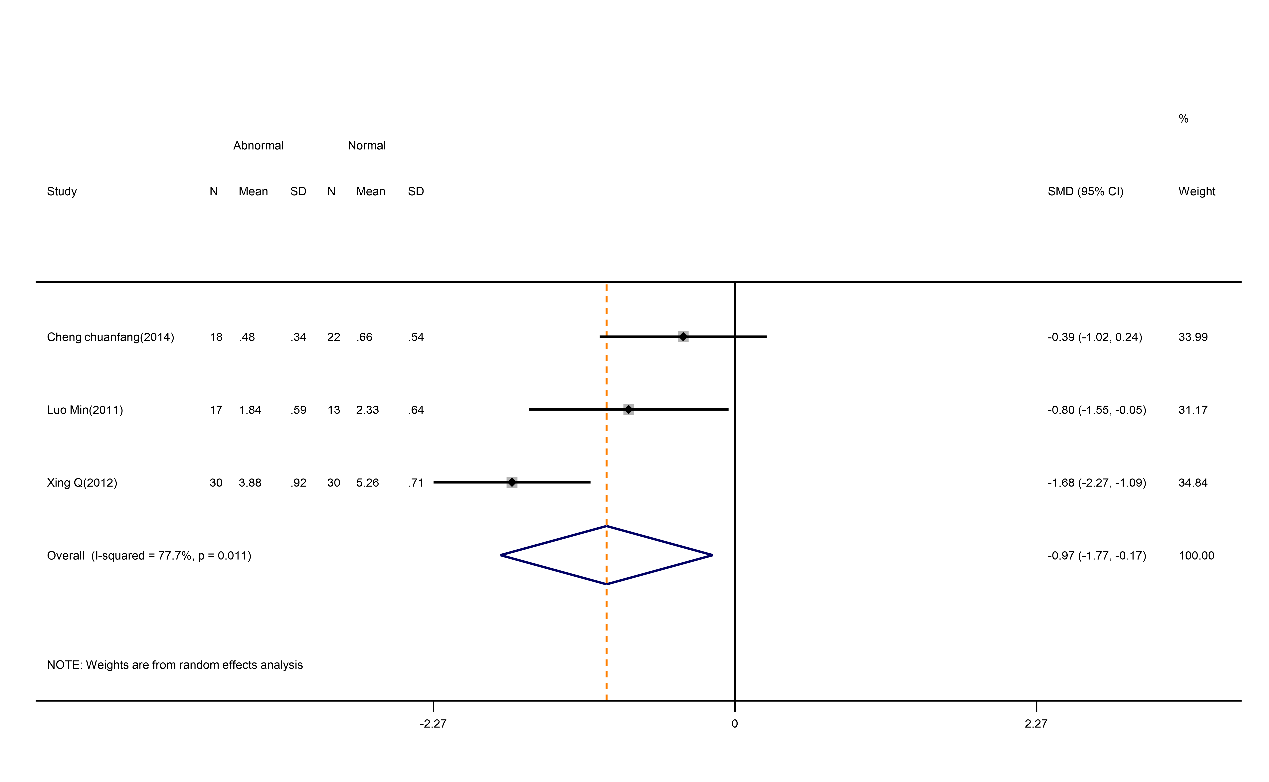


Supplementary Fig.D. 9 Subgroup analysis in Th17 cells according to kidney function of SLE patients. Abnormal: SLE patients with abnormal kidney function (El-Maraghy N (2018) was removed). Normal: SLE patients with normal kidney function.


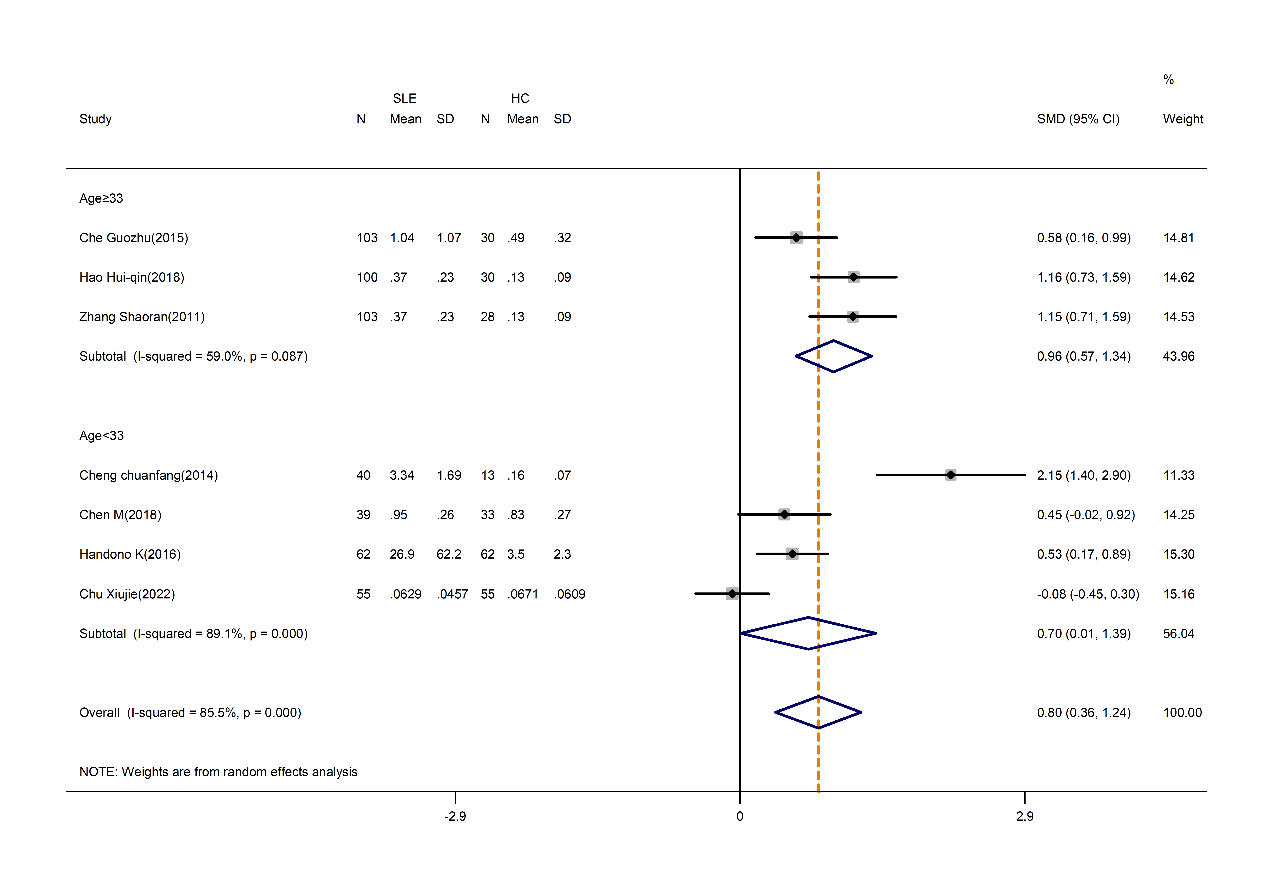


Supplementary Fig.D. 10 Subgroup analysis in the ratio change of Th17/Treg cells according to the patient age.


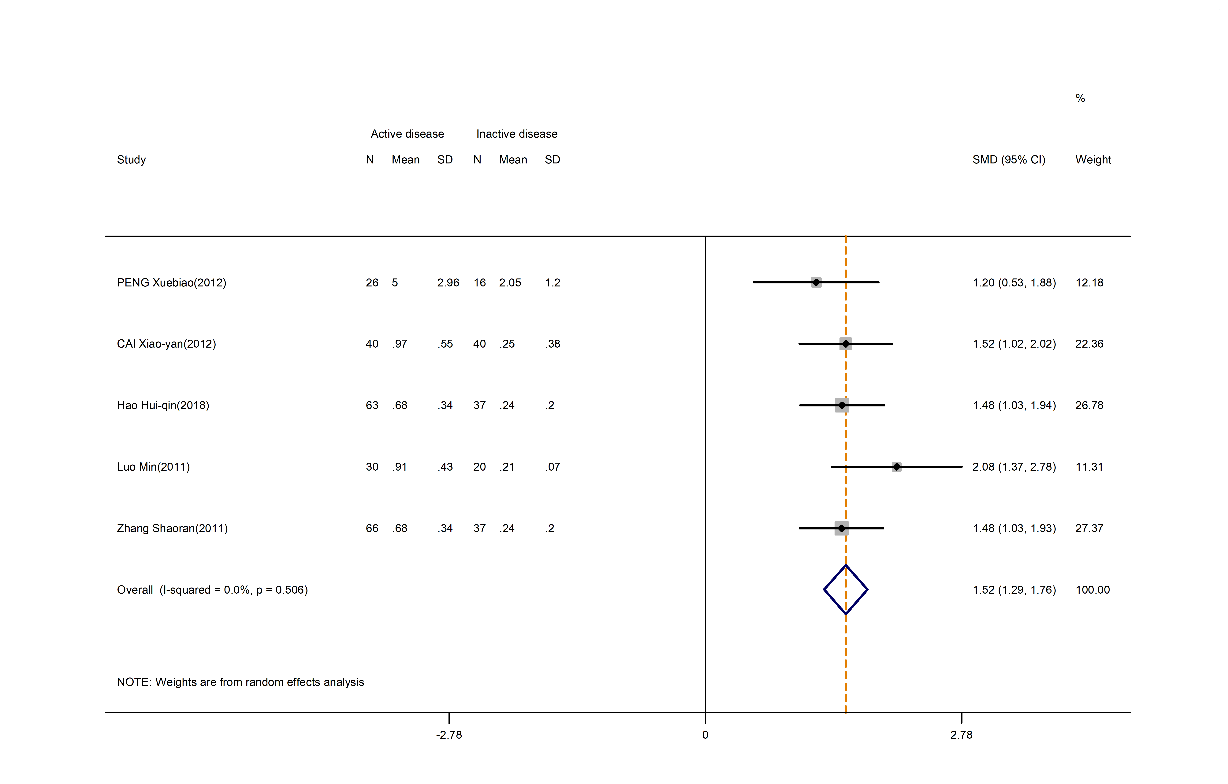


Supplementary Fig.D. 11 Forest plot of the ratio change of Th17/Treg cells in patients with SLE in active disease vs. inactive disease (Che Guozhu (2015) was removed).


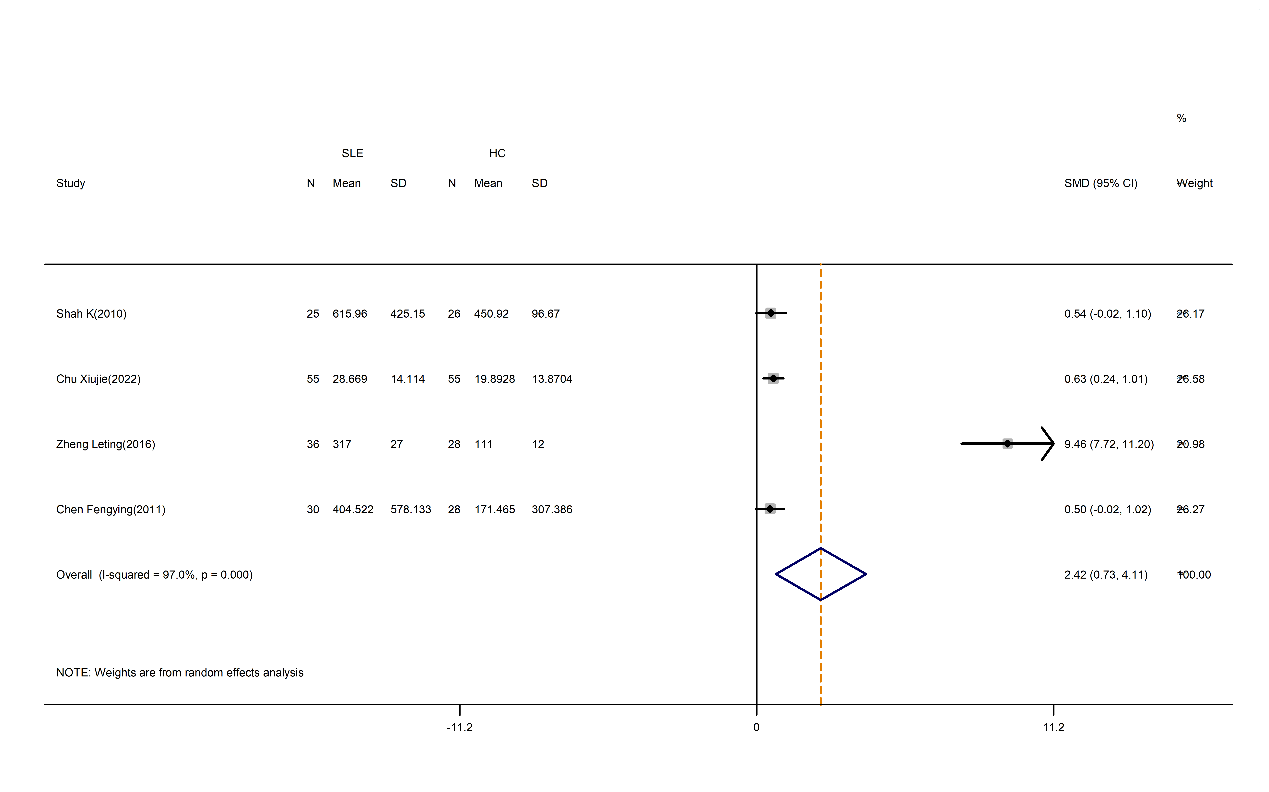


Supplementary Fig.D. 12 Forest plot of the level of IL-21 in SLE patients compared with HCs.

Supplementary Fig.D. 13 Forest plot of the level of IL-17 in SLE patients compared with HCs (Xing Q (2012) and Yang XY (2013) were removed).


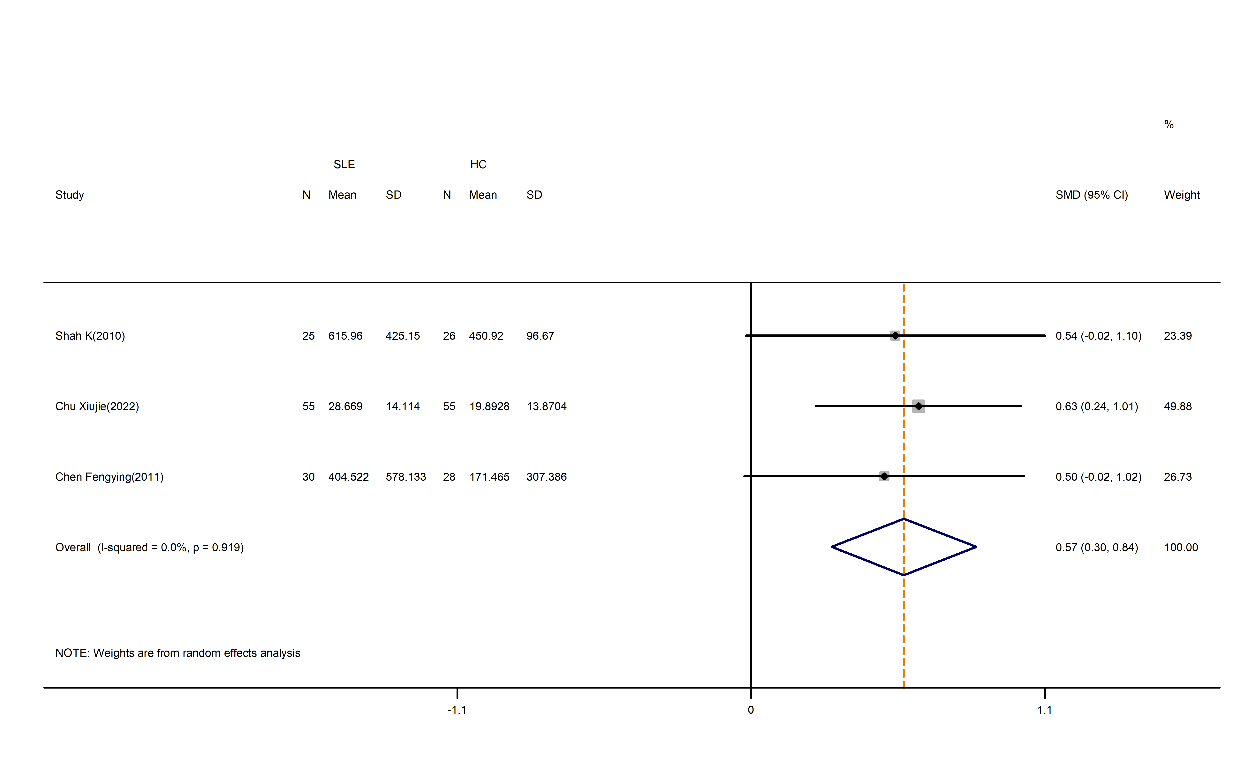


Supplementary Fig.D. 14 Forest plot of the level of IL-21 in SLE patients compared with HCs (Zheng Leting (2016) was removed).


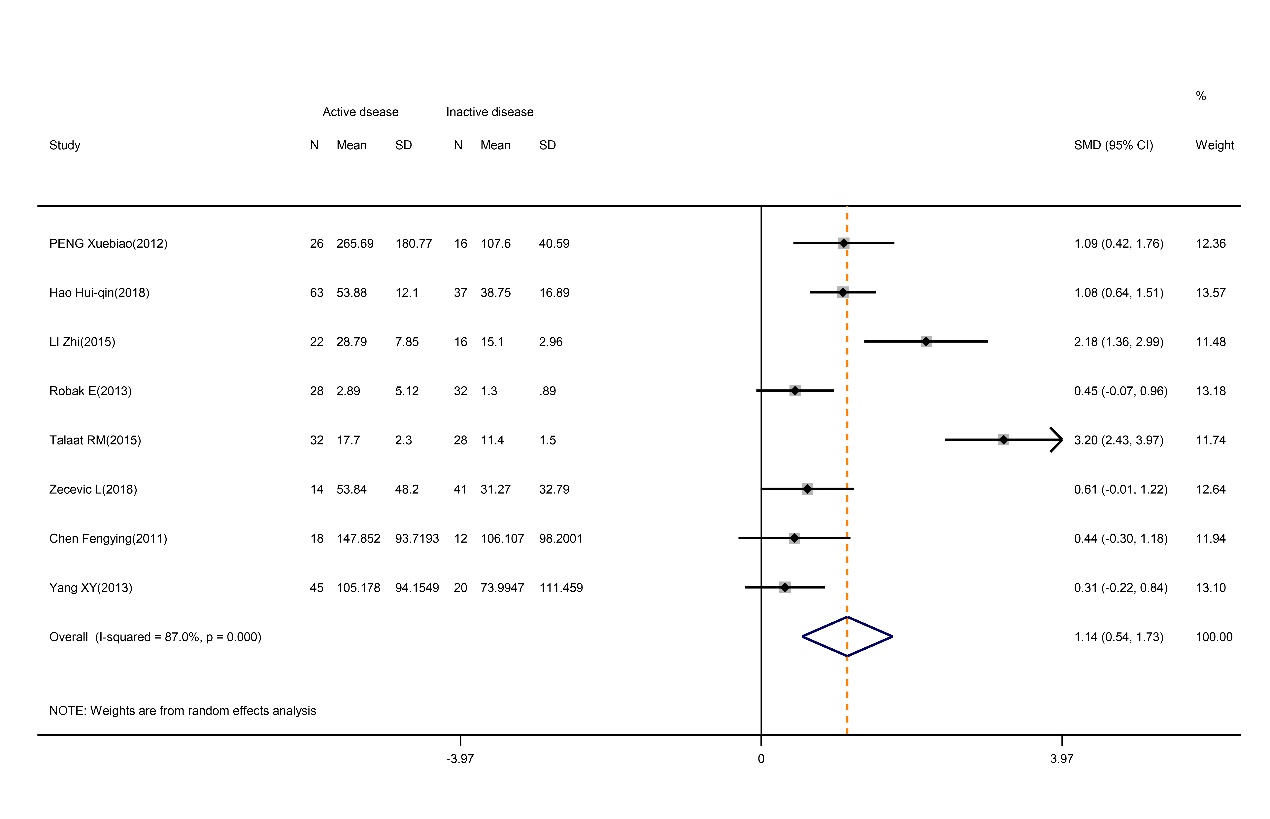


Supplementary Fig.D. 15 Forest plot of the level of IL-17 in patients with SLE in active disease vs. inactive disease.


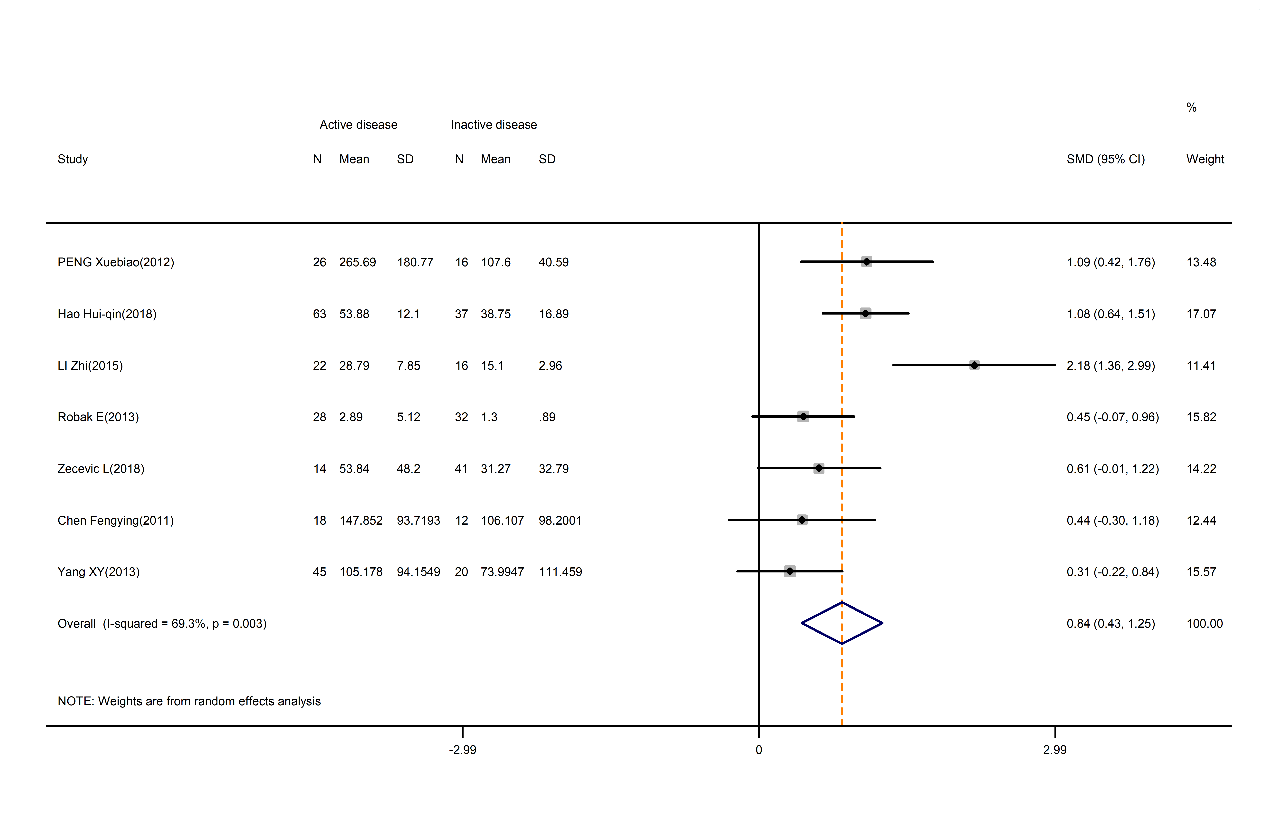


Supplementary Fig.D. 16 Forest plot of the level of IL-17 in patients with SLE in active disease vs. inactive disease (Talaat RM (2015) was removed).


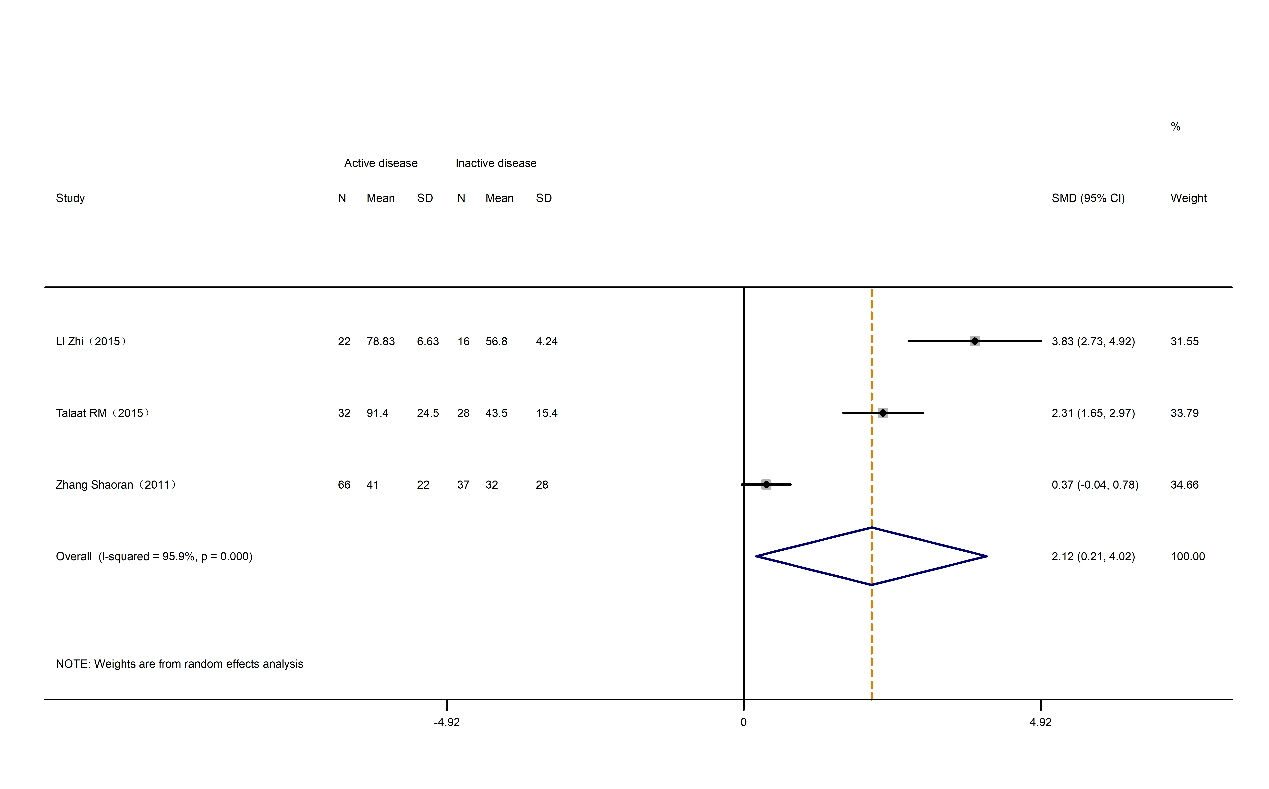


Supplementary Fig.D. 17 Forest plot of the level of IL-6 in patients with SLE in active disease vs. inactive disease.


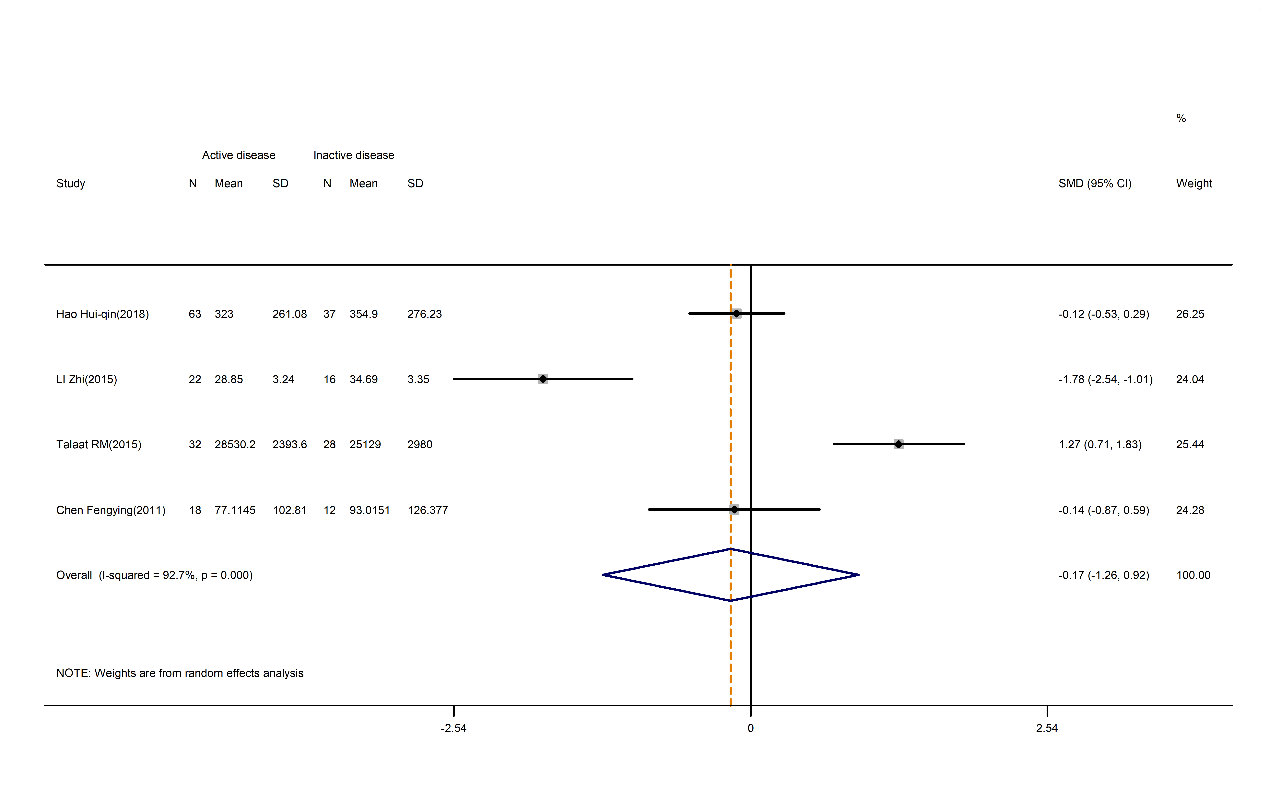


Supplementary Fig.D. 18 Forest plot of the level of TGF-β in patients with SLE in active disease vs. inactive disease.


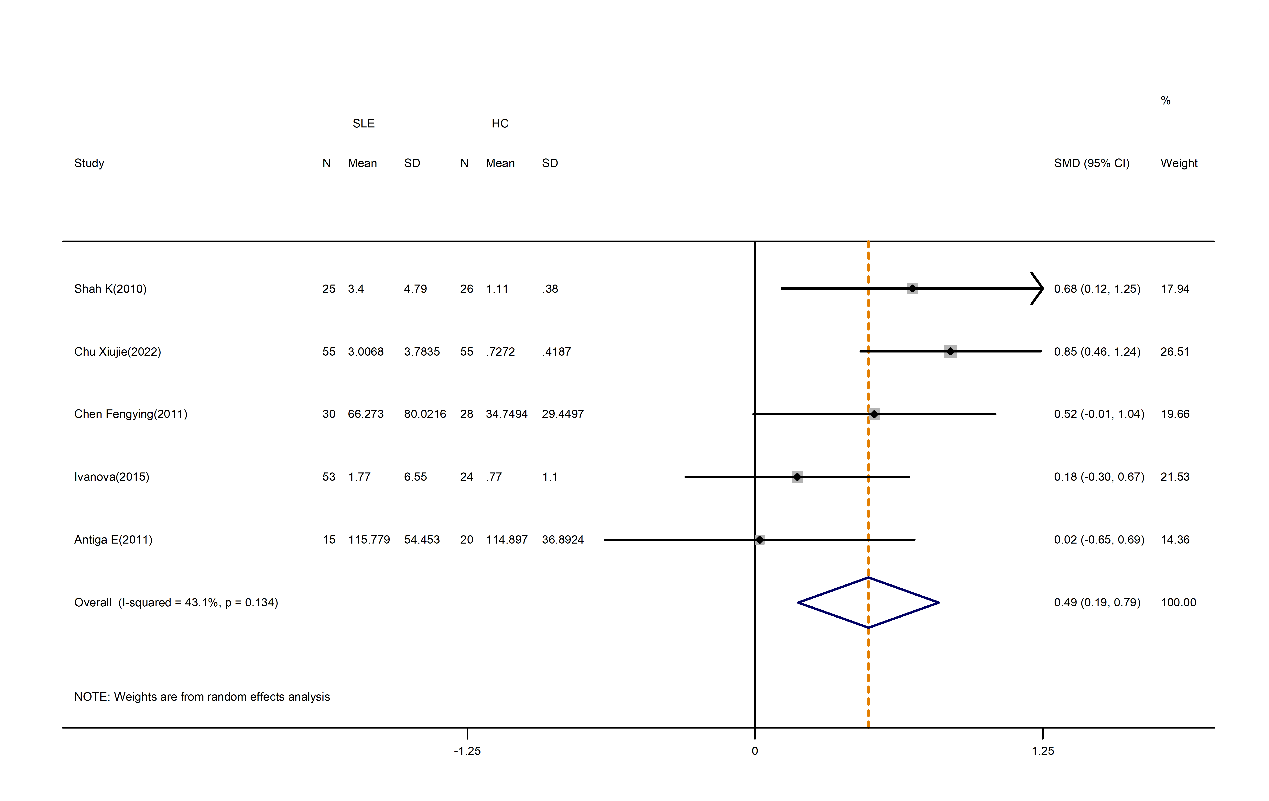


Supplementary Fig.D. 19 Forest plot of the level of IL-10 in SLE patients compared with HCs.


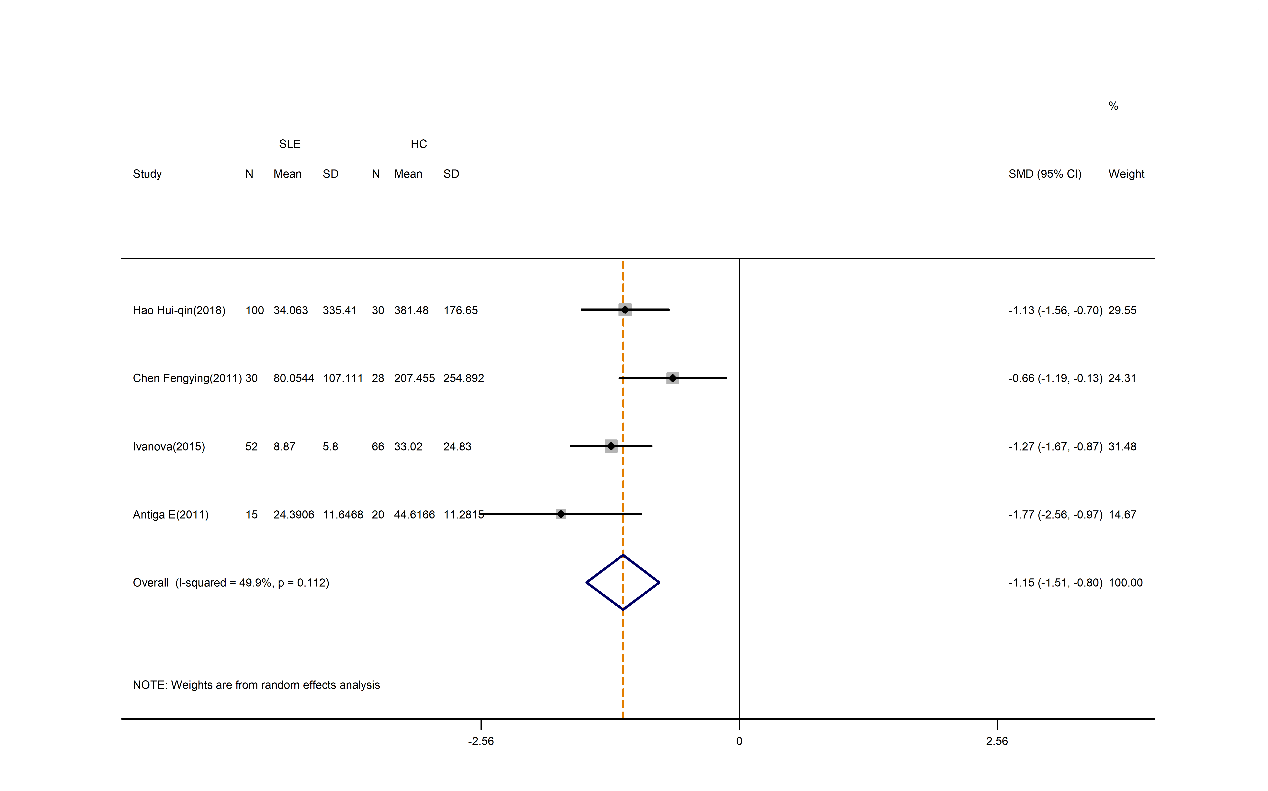


Supplementary Fig.D. 20 Forest plot of the level of TGF-β in SLE patients compared with HCs (Xing Q (2012) was removed).


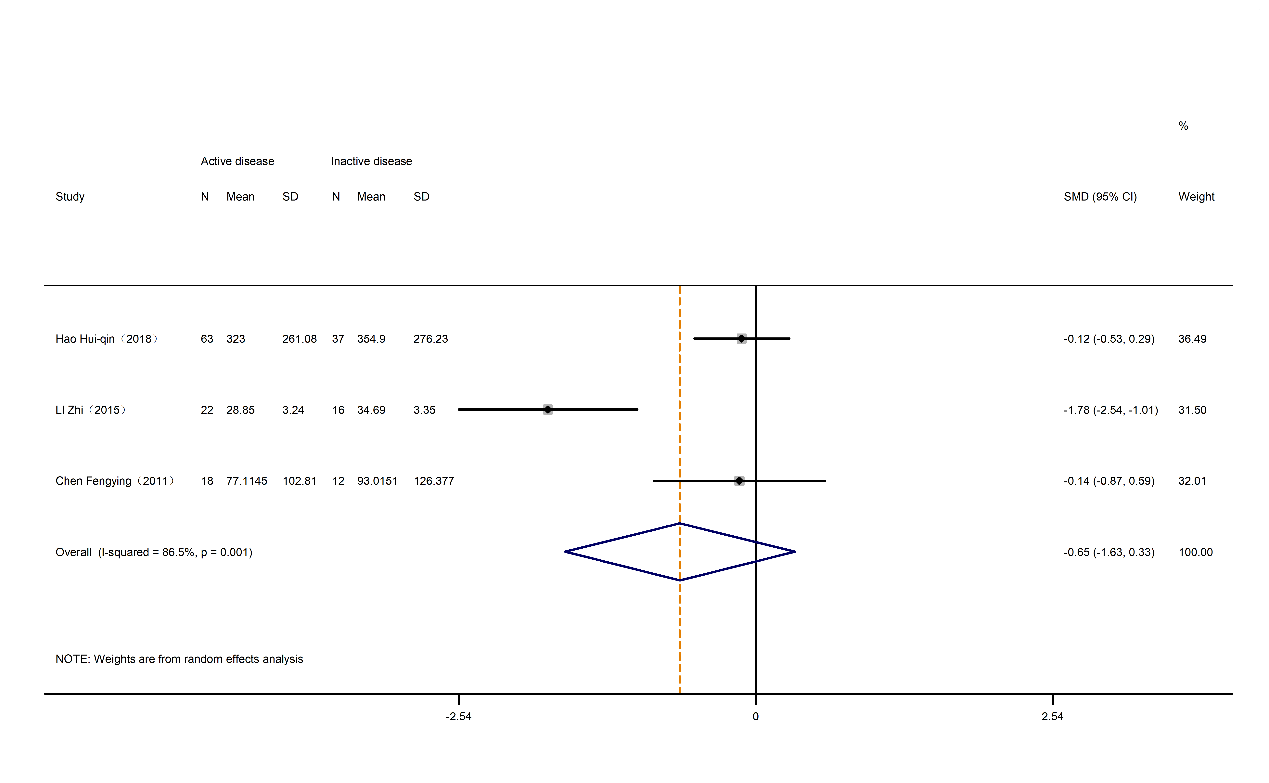


Supplementary Fig.D. 21 Forest plot of the level of TGF-β in patients with SLE in active disease vs. inactive disease (Talaat RM (2015) was removed).
